# Effect of porcine corneal stromal extract on keratocytes from SMILE‐derived lenticules

**DOI:** 10.1111/jcmm.16189

**Published:** 2020-12-20

**Authors:** Shenyang Li, Zekai Cui, Jianing Gu, Yini Wang, Shibo Tang, Jiansu Chen

**Affiliations:** ^1^ Aier School of Ophthalmology Central South University Hunan China; ^2^ Aier Eye Institute Changsha China

**Keywords:** human corneal stromal cells, intrastromal injection, porcine corneal stroma extract, serum‐low RIFA medium, SMILE‐derived lenticule

## Abstract

Propagating large amounts of human corneal stromal cells (hCSCs) in vitro while maintaining the physiological quality of their phenotypes is necessary for their application in cell therapy. Here, a novel medium to propagate hCSCs obtained from small incision lenticule extraction (SMILE)‐derived lenticules was investigated and the feasibility of intrastromal injection of these hCSCs was assessed. Primary hCSCs were cultured in porcine corneal stroma extract (pCSE) with RIFA medium including ROCK inhibitor Y27632, insulin‐transferrin‐selenium, fibroblast growth factor 2, L‐ascorbate 2‐phosphate and 0.5% FBS (RIFA medium + pCSE). Protein profiling of the pCSE was identified using nanoscale liquid chromatography coupled to tandem mass spectrometry (nano LC‐MS/MS). After subculturing in RIFA medium + pCSE or 10% FBS normal medium (NM), hCSCs at P4 were transplanted into mouse corneal stroma. Compared with NM, ALDH3A1, keratocan and lumican were significantly more expressed in the RIFA medium + pCSE. ALDH3A1 was also more expressed in the RIFA medium + pCSE than in the RIFA medium. Fibronectin and α‐SMA were less expressed in the RIFA medium + pCSE than in the NM. Using Metascape analysis, the pCSE with its anti‐fibrosis, pro‐proliferation and anti‐apoptosis activities, was beneficial for hCSC cultivation. The intrastromally implanted hCSCs in the RIFA medium + pCSE had positive CD34 expression but negative CD45 expression 35 days after injection. We provide a valuable new medium that is advantageous for the proliferation of hCSCs with the properties of physiological keratocytes. Intrastromal injection of hCSCs in RIFA medium + pCSE has the potential for clinical cell therapy.

## 1 INTRODUCTION

The corneal stroma is mainly composed of collagen and corneal stromal cells (CSCs). The physiological CSCs (also called keratocytes) present the characteristics of quiescent and dendritic cells. They usually express the cluster of differentiation 34 (CD34), stromal crystallins (ALDH3A1) and keratan sulphate proteoglycans (KSPGs), including lumican, keratocan and mimecan, which contribute to corneal transparency.^1^ Keratocytes synthesize collagens, other extracellular matrix (ECM) elements, and various enzymes to degrade old matrix proteins to maintain stromal matrix homeostasis.^2^ Keratocytes can be transformed into motile and contractile fibroblasts or myofibroblasts largely due to the activation of the transforming growth factor‐β (TGF‐β) system during stromal damage and the healing process.^3^ Myofibroblasts express high levels of alpha‐smooth muscle actin (α‐SMA), biglycan and the extra domain A (EDA) of cellular fibronectin, which usually causes corneal scarring.^4^ Corneal disease is one of the major leading causes of blindness globally.^5^ Corneal transplantation is the definitive treatment for patients with severe corneal lesions.^6^ However, the clinically successful application of corneal transplantation is limited due to the lack of donated corneal tissue and post‐transplant complications.^7^, ^8^ Thus, developing alternatives is imperative.

Cell therapy using healthy CSCs to replace diseased stromal cells is an available approach. Propagating large amounts of human CSCs in vitro while maintaining the physiological quality of their phenotypes is necessary for their application in cell therapy. However, when cultured in serum‐containing medium, CSCs easily transform and can lose important cell properties after a limited scale of cell expansion in vitro.^9^ Culture substrate/scaffold conditions, cell densities and other elements also influence keratocyte‐myofibroblast transdifferentiation in in vitro cultures.^10^, ^11^ Although serum‐free cultures have been reported to be effective for the maintenance of the phenotype and physiological properties of keratocytes, the subcultivation of a large amount of CSCs remains challenging.^12^ Various improved culture techniques have been reported, including spheroid cultures,^13^ media supplements,^2^ derivation from human stem cells^14^ and culturing on special materials.^10^, ^11^ In terms of medium supplements, extracts from cells or tissues have also been applied. Several studies demonstrated that extracts obtained from mammalian and embryonic stem cells (ESCs) had beneficial effects on cellular reprogramming.^15^, ^16^ Amniotic membrane extract (AME) has also been successfully used as an eye drop in clinical applications to treat dry eye and chemical burns.^17^ Yam et al^2^ used cocktail medium supplemented with soluble AME, ROCK inhibitor Y‐27632, and insulin‐like growth factor‐1 (IGF‐1) to propagate and maintain CSCs. They reported that this type of medium supplementation promoted the keratocyte features and prevented keratocyte‐myofibroblast transdifferentiation. Proteins > 3 kDa in soluble AME enhanced keratocyte growth and inhibited cell fibrosis.

Small incision lenticule extraction (SMILE) is a corneal refractive surgical procedure used to correct myopia or other refractive errors. With increasing numbers of patients undergoing SMILE, there are many extracted pieces of intrastromal lenticule, which are usually discarded. SMILE‐derived lenticules were recently successfully used in both preclinical animal studies and human clinical applications. Clinical studies have demonstrated that corneal intrastromal lenticules can be successfully reutilized to treat high hyperopia and presbyopia,^18^, ^19^ thin corneas due to recurrent pterygium,^20^ keratoconus,^21^, ^22^ corneal ulcer or perforations,^23^ and other corneal diseases.^24^ Our prior research demonstrated that SMILE‐derived lenticules were beneficial scaffolds for the reconstruction of retinal pigment epithelial (RPE) sheets. Lenticules displayed biocompatibility after subretinal implantation.^25^ Therefore, discarded corneal lenticules from SMILE can be reused as repair biomaterials or scaffolds for tissue engineering. Fresh lenticules can also be used as sources to acquire human CSCs (hCSCs) in vitro although no reports have been documented in the literature. Mimura et al reported that both the peripheral and central regions of rabbit corneal stroma contained a significant number of precursors using spherical cultivation, but the peripheral stroma had more precursors with a stronger proliferative capacity than cells from the central stroma.^26^ Therefore, improving the quantity and quality of expanded and activated cells for cell therapy when using primary cells from SMILE‐derived lenticules of central stroma rather than the peripheral region is necessary.

This study aimed to develop an effective approach for the proliferation of hCSCs with preferable cell viability and physiological properties. We used soluble porcine corneal stromal extract (pCSE) with low‐serum RIFA medium primarily including ROCK inhibitor Y27632, insulin‐transferrin‐selenium (ITS), fibroblast growth factor 2 (FGF2), L‐ascorbate 2‐phosphate and 0.5% foetal bovine serum (FBS) to culture primary hCSCs obtained from SMILE‐derived lenticules. This study provides insights into the probable mechanisms of pCSE’s effect on hCSCs by analysing the protein composition using nano LC‐MS/MS. We also assessed the applicability of injecting hCSCs into mouse corneas and explored the effects. We determined whether using pCSE with low‐serum RIFA medium not only promoted hCSC proliferation, but also maintained the physiological function of human keratocytes. We also assessed the potential of using hCSCs from SMILE‐derived lenticules for corneal stromal cell therapy.

## 2 MATERIALS AND METHODS

### 2.1 Ethics statement

Porcine ocular tissues were obtained from a slaughterhouse and the pigs were certified by the Animal Quarantine Bureau of China. The use of human tissue samples was approved by the ethics committee of Aier Eye Hospital (Changsha, Hunan, China), and the methods for securing human tissues were in compliance with the Declaration of Helsinki. Informed consent was obtained from all patients. Female C57BL/6 mice, 8‐10 weeks of age, were supplied by the Third Xiangya Hospital of Central South University. All animal experiments were approved by the Animal Ethics Committee of the Central South University and were in accordance with the National Institutes of Health Guide for the Care and Use of Animals and the Association for Research in Vision and Ophthalmology (ARVO) statement for the use of animals in ophthalmic and vision research.

### 2.2 Preparation of soluble porcine corneal stromal extract (pCSE)

The pCSE was prepared with reference to published methods reported by Yam GH et al^2^ Briefly, porcine corneas (n = 8‐10, either gender) were isolated from fresh porcine eyes. After they were rinsed with sterile saline containing gentamicin and the epithelial and endothelial layers were removed, the tissues were used to prepare pCSE. The stromal layers were devitalized in DMEM/F12‐glycerol (50:50 vol/vol) at −80°C. After they were rinsed with phosphate‐buffered saline (PBS), the corneal stromal layers were drip‐dried, weighed, and ground under the air phase of liquid nitrogen. The homogenate was suspended in ice‐cold sterile PBS (5 ml per gram of tissue). The subsequent steps were performed at 4°C. The suspension was rotated at 300 rpm (THZ‐C‐1; Taichang, Changsha, China) for 48 h and centrifuged at 15,000 g for 20 min to remove insoluble debris. The supernatant was further filtered using a 0.22 μm membrane filter. Sterile pCSE was collected and stored at −80°C for further use. The total protein concentration was determined via a BCA assay (SolarBio, Beijing, China).

### 2.3 Live/Dead viability assay

The live/dead assay was used to examined hCSC viability of lenticules after suffering femtosecond laser irradiation and was performed according to the manual of the Live/Dead Viability Assay Kit (Beyotime, Shanghai, China). Then, images were captured by an LSM800 confocal microscope (Zeiss, Germany).

### 2.4 Isolation and culture of human corneal stromal cells

The human corneal stromal cells (hCSCs) were isolated from SMILE‐derived lenticules (Changsha Aier Eye Hospital, Changsha, China). A total of 20 stromal lenticules were obtained from donor myopic patients, whose age was ranged from 22 to 32 years old. The central thickness of lenticules was 60‐150 μm, and the peripheral thickness was 10‐30 μm. The lenticules were rinsed in sterile PBS and digested with 1 mg/ml collagenase I (Sigma‐Aldrich, St. Louis, MO, USA) for 6 to 8 h at 37°C. The mixture was then centrifuged to collect cells. After centrifuging, the pellets were suspended in 5 μg protein/ml soluble pCSE with low‐serum RIFA medium including DMEM/F12 (Gibco, Gaithersburg, MD, USA), 10 μM ROCK inhibitor Y27632 (AdooQ Bioscience, Irvine, CA, USA), 10 ng/ml ITS (Life Technologies, Carlsbad, CA, USA), 10 ng/ml FGF2 (PeproTech, Rocky Hill, NJ, USA), 1 mM L‐ascorbate 2‐phosphate (Sigma, St. Louis, MO, USA), 0.5% FBS (Gibco, Gaithersburg, MD, USA) and 1% penicillin‐streptomycin (P/S, Gibco, Gaithersburg, MD, USA). The cells were seeded on collagen‐coated cell plates with a concentration of 4 × 10^4^ cells/ml. The plates were incubated at 37℃ in a humidified atmosphere at 5% CO_2,_ and the media were changed every 2‐3 days. Confluent cell layers were dissociated and subcultured at the same seeding density with RIFA medium supplemented with pCSE or normal medium (NM) which comprised DMEM/F12 supplemented with 10% FBS and 1% penicillin‐streptomycin. Only hCSCs at passage four (P4) were used in this study.

### 2.5 Anti‐fibrosis assay

The hCSCs at P4 in RIFA medium supplemented with pCSE were seeded in 6 well plates at a density of 1 × 10^4^cells/well and allowed to attach for 24 hours. The cells were then rinsed with PBS three times, split into six groups: (1) RIFA medium + 0μg/ml pCSE + 10ng/ml TGFβ1, (2) RIFA medium + 0.05μg/ml pCSE + 10ng/ml TGFβ1, (3) RIFA medium + 0.5μg/ml pCSE + 10ng/ml TGFβ1, (4) RIFA medium + 5μg/ml pCSE + 10ng/ml TGFβ1, (5) RIFA medium + 50μg/ml pCSE + 10ng/ml TGFβ1, and (6) RIFA medium + 5μg/ml pCSE. Media were changed every third days and every media had freshly prepared TGFβ1 added to ensure similar TGFβ1 activity throughout the experiment. Cells in different culture conditions were collected at 7 days.

### 2.6 Immunofluorescence (IF)

After fixation, permeabilizing and blocking, cells at P4 were then incubated overnight at 4℃ with primary antibodies, including rabbit anti‐ALDH3A1(1:200, Abcam), rabbit anti‐lumican (1:400, Abcam), rabbit anti‐Fibronectin (1:200, Abcam) and rabbit anti‐α‐SMA (1:500, Abcam) followed by washing three times in PBS and incubation with FITC‐conjugated anti‐rabbit and Cy3‐conjugated anti‐rabbit IgG secondary antibodies (1:500, Life) for 1 h at room temperature and were incubated with DAPI for nuclear staining for 15 min. Finally, the imaging was performed using an LSM800 confocal microscope (Zeiss, Germany).

### 2.7 Quantitative polymerase chain reaction (qPCR)

Total RNA from hCSCs was extracted using the High Pure RNA Isolation Kit (Roche). The cDNA was synthesized using Revert Aid First Strand cDNA Synthesis Kit (Thermo Scientific) according to the manufacturer's instructions. Gene‐specific primers were synthesized by TsingKe Biotech (China), and the sequences of the primers are listed in Supplementary Table 1 (Table S1). For qPCR experiments, gene expression was analysed by qPCR (Roche) with three replicates per sample. The results of amplification were normalized to GAPDH mRNA transcript. Expression changes in the gene transcripts for each sample were calculated using the 2^‐△△Ct^ method. The results from three independent experiments were statistically analysed.

### 2.8 Western blotting assay

The total hCSC proteins were extracted and the protein concentrations were detected using a BCA Protein Assay Kit (Solarbao, China). The protein samples were run through sodium dodecylsulphate‐polyacrylamide gel electrophoresis (SDS‐PAGE) and transferred to polyvinylidene fluoride (PVDF) films. After blocking, the membranes were incubated with antibodies, including rabbit anti‐ALDH3A1(1:500, Abcam), rabbit anti‐keratocan (1:100, Abcam), rabbit anti‐lumican (1:1000, Abcam), rabbit anti‐fibronectin (1:1000, Abcam), rabbit anti‐α‐SMA (1:1000, Abcam) and mouse anti‐GAPDH overnight at 4℃. The membranes were then incubated with anti‐mouse or anti‐rabbit IRDye 680RD secondary antibodies (1:10 000, LI‐CORBiosciences) for 2 h at room temperature. Bands were visualized with the Odyssey Fc Imaging System (LI‐COR Biosciences, USA) and quantified with Image J software. The expression ratios of the target proteins were determined after normalizing the individual GADPH levels. The results from three independent experiments were statistically analysed.

### 2.9 5‐ethynyl‐2′‐deoxyuridine (EdU) labelling assay

The hCSCs at P4 in RIFA medium supplemented with pCSE were seeded on 48‐well plates at 1 × 10^4^ cells/well and continually cultured in the RIFA medium, RIFA medium supplemented with pCSE or NM for another 2 days. The EdU labelling assay was conducted according to the manual of the EdU labelling/detection kit (Keygen, Jiangsu, China). Samples were then observed and photographed under a fluorescence microscope. The percentage of EdU‐positive cells was calculated using ImageJ software, respectively, from six random fields in three random wells.

### 2.10 Cell Counting Kit‐8 (CCK‐8) assay

CCK‐8 was used to detect the cell viability of hCSCs cultured in RIFA medium, RIFA medium supplemented with pCSE or NM. Briefly, hCSCs at P4 were seeded at 3000 cells/well in eight 96‐well plates and treated with different media for 0‐7 d, respectively. Then, cells from one plate were stained with 10 μl CCK‐8 (Solarbio, Beijing, China) solution and incubated at 37℃ for 2 h every day. Absorbance at 450 nm was measured with a microplate reader (BioTek, VT, USA). Six parallel experiments in each sample were used and three independent experiments were statistically analysed.

### 2.11 Phycoerythrin (PE) Annexin V and 7‐Amino‐Actinomycin (7‐AAD) assay

The hCSCs at P4 in RIFA medium supplemented with pCSE were seeded into 6‐well plates at the density of 1 × 10^5^ cells/well in the RIFA medium or RIFA medium supplemented with pCSE for 24 h. To establish an oxidative stress model, 500 μM hydrogen peroxide (H_2_O_2_) was added to the culture medium for 24 h at 37 ℃, 5% CO_2_ incubator. Apoptosis staining was performed using a PE Annexin V Apoptosis Detection Kit I as the manufacturer's instructions and stained cells were instantly measured using flow cytometry (BD FACSCelesta, USA). One hundred thousand events were collected for each sample.

### 2.12 Nanoscale liquid chromatography coupled to tandem mass spectrometry (Nano LC‐MS/MS) assay, enzyme‐linked immunosorbent assay (ELISA) and bioinformatic analyses

The three independent pCSE samples were prepared. The nano LC‐MS/MS and ELISA assays were subcontracted to Beijing Biotech‐Pack Scientific (Beijing, China). The details are described in Supplementary Methods 1‐4. The identified proteins’ biological processes were analysed using Metascape.^27^

### 2.13 Intrastromal injection of hCSCs

On the transplantation day, the P4 cells in RIFA medium supplemented with pCSE and NM were digested and passed through a cell strainer to obtain single‐cell suspensions. After they were washed, the total cell count was measured and living cells were counted using a trypan blue exclusion assay. The hCSCs were labelled with PKH26 (Sigma‐Aldrich, St. Louis, MO, USA) following the manufacturer's protocol and washed twice in fresh media. The cells were suspended in DMEM/F12 at a concentration of 1.25 × 10^4^ cells per microlitre before injection. After general anaesthesia and the topical application of oxybuprocaine hydrochloride eye drops (Santen Pharmaceutical Co., Ltd., Chongqing, China), an intrastromal tunnel was produced in the mouse anterior corneal stroma using a 33‐gauge needle. 2.5 × 10^4^ cells in 2 μl DMEM/F12 were injected into the stroma through a blunt 33‐gauge needle attached to a Hamilton syringe (Hamilton Company, Reno, NV, USA). Ofloxacin eye drops (Santen Pharmaceutical Co., Ltd., Chongqing, China) were then applied to the eyes.

### 2.14 Ophthalmic examination and measurements

The injected corneas were divided into three groups: a RIFA medium supplemented with pCSE group, an NM group and a blank group. Corneal cross‐section visualization and measurement of the mean corneal thickness (MCT) were performed using anterior segment optical coherence tomography (AS‐OCT, Phoenix Micron IV; Phoenix Technology Group, Pleasanton, CA, USA). The corneal imaging and measurements were performed 7 days prior to injection. All of the corneas were examined centrally (six mice for each group at 0, 7, 21 and 42 days). The measurements were conducted three times in each eye, and the average of the three readings was used. The post‐injection and pre‐injection MCT were measured using ImageJ software. To evaluate changes in the anterior segment, slit‐lamp photographs of six mice in each group were obtained using a zoom photograph slit lamp (Nikon FS‐3V; Nikon, Tokyo, Japan) 35 days after injection. All of the corneas injected with PKH26‐labelled cells were mounted on a slide for confocal laser scanning microscopy. For immunostaining, frozen sections (10 μm) were stained with CD34 and CD45, respectively. 4',6‐diamidino‐2‐phenylindole (DAPI) was used to stain the nuclei. Images were obtained via confocal laser scanning microscopy. For whole‐mount immunostaining, the corneas were incubated with primary antibody followed by incubation with fluorescence‐conjugated secondary antibody. The corneas were then mounted on slides for confocal laser scanning microscopy.

### 2.15 Statistical analysis

Values are expressed as the mean ± SD of values obtained from three or more samples. Statistical analysis between two groups was carried out using Student's unpaired t test; comparison among multiple groups was determined by one‐way ANOVA *P* < .05 was considered to be statistically significant.

## 3 RESULTS

### 3.1 Isolation of hCSCs from SMILE‐derived lenticules and culture in RIFA medium supplemented with pCSE (RIFA medium + pCSE)

**FIGURE 1:**
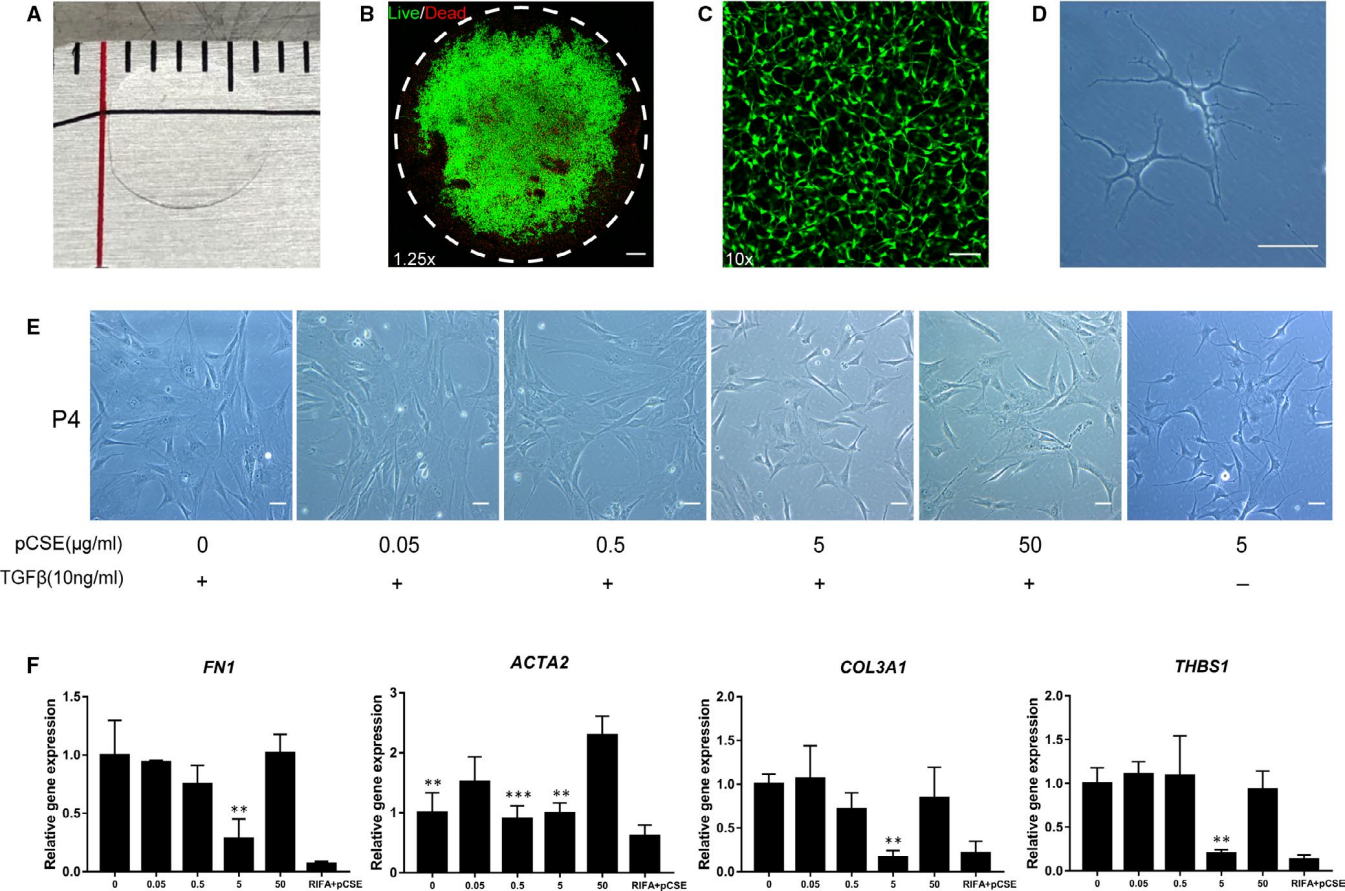
The characteristic of SMILE‐derived lenticule and screening optimal pCSE anti‐fibrotic concentration in the low‐serum RIFA medium using TGFβ1‐induced fibrosis assay. (A) Photograph of a SMILE‐derived lenticule. (B, C) The results of hCSC live/dead staining in lenticules are shown. (D) Under light microscopy, the morphology of primary keratocytes. (E) Under light microscopy, the cells demonstrated multiple morphological characteristics, such as slender, spindle or polygonal shapes, while only the cells in the 5 μg/ml group were small and stellate shaped. In contrast, the hCSCs cultured in the RIFA medium supplemented with pCSE (RIFA medium + pCSE) had dendritic or stellate shapes. (F) RT‐qPCR analysis indicated that the fibrotic genes were lowest in the 5 μg/ml pCSE among the concentrations (**P < .01; ***P < .001). Scale bars: 500 μm for B; 100μm for C; 50 μm for D and E

The hCSCs were successfully isolated from SMILE‐derived lenticules (Figure 1A) using collagenase I digestion (about 1 × 10^4^ cells from each lenticule). The live/dead staining showed that most of the live cells were located in the centre of the lenticules, while the cells in the peripheral region were dead (Figure 1B, 1C). In the primary culture, the hCSC monolayer reached confluence within 5‐7 days in the RIFA medium supplemented with pCSE. The hCSCs exhibited dendritic or stellate shapes, and cell processes extended to connect with neighbouring cells (Figure 1D). The results indicated that SMILE‐derived lenticules can be used as a source of hCSCs, demonstrating their physiological cell morphology in RIFA medium supplemented with pCSE.

### 3.2 Optimal concentration of pCSE in RIFA medium for the growth of hCSCs

We explored the optimal concentration of pCSE in RIFA medium using a qPCR assay. The hCSCs at passage 4 (P4) were cultured in different concentration groups (0 μg/ml, 0.05 μg/ml, 0.5 μg/ml, 5 μg/ml and 50 μg/ml) of pCSE in RIFA medium and treated with 10 ng/ml TGFβ1 for 7 days. As demonstrated by light microscopy, the hCSCs expanded considerably under these conditions. The cells had many morphological characteristics, such as slender, spindle and polygonal shapes. Cells had small and stellate shapes only in the 5 μg/ml group. In contrast, the hCSCs cultured in RIFA medium supplemented with pCSE had dendritic or stellate shapes (Figure 1E). The qPCR analysis indicated that fibrotic genes *FN1*, *ACTA2*, *COL3A1* and *THBS1* were low in the 5 μg/ml pCSE group at all of the concentrations (Figure 1F). Based on these results, 5 μg/ml pCSE in RIFA medium is an optimal concentration for hCSC cultures and was used in later studies.

### 3.3 Characteristics of the hCSCs cultured in low‐serum RIFA medium supplemented with pCSE

**FIGURE 2:**
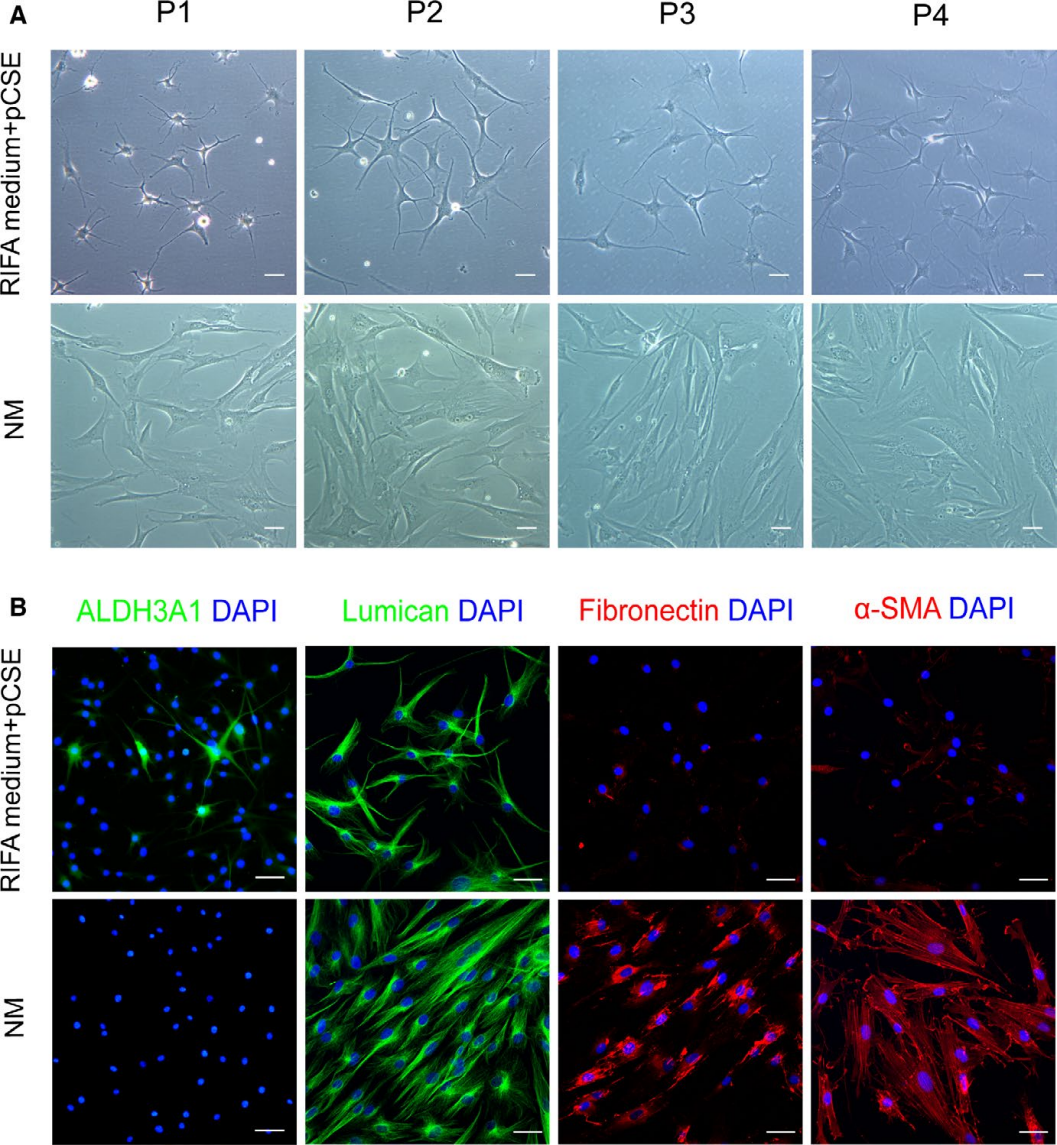
Characteristics of adherent hCSCs cultured in the RIFA medium + pCSE and normal medium (NM) groups. (A) The hCSCs at P1‐P4 had differing morphologies. (B) Immunofluorescence image of ALDH3A1, lumican, fibronectin and α‐SMA of the hCSCs at P4. Scale bars: 50 μm

**FIGURE 3:**
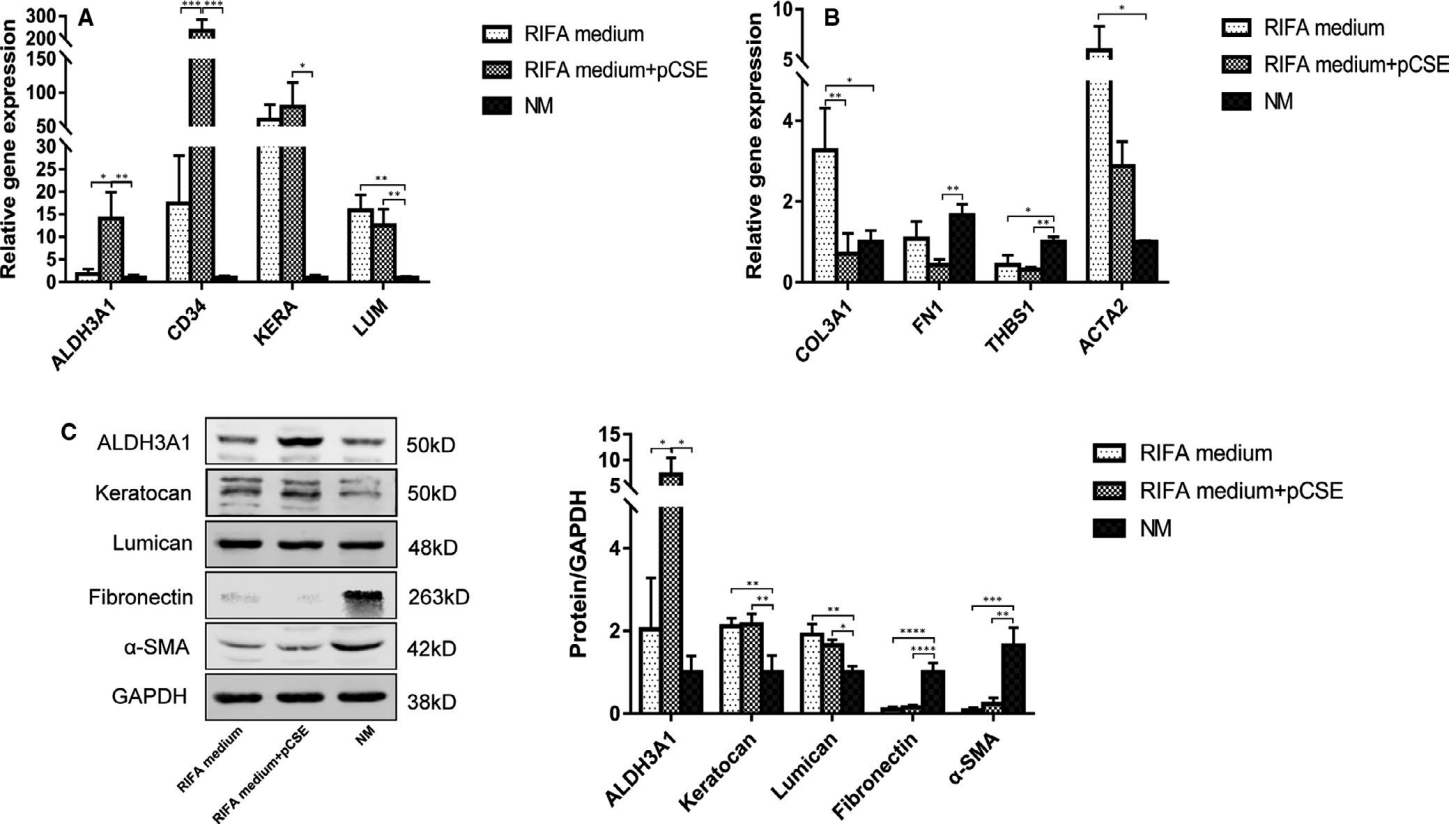
The maintenance of the hCSC phenotype after treatment with RIFA medium + pCSE. (A) RT‐qPCR analysis showed that the keratocyte marker‐related genes *ALDH3A1*, *CD34*, *KERA* and *LUM* were significantly greater in the RIFA medium + pCSE group than in the NM group. *ALDH3A1* and *CD34* were also up‐regulated in the RIFA medium + pCSE group compared with the RIFA medium group. (B) The fibrotic genes (*FN1* and *THBS1*) were significantly lower in the RIFA medium + pCSE group than in the NM group. Other fibrotic genes (*COL3A1* and *ACTA2*) were significantly higher in the RIFA medium group than in the RIFA medium + pCSE and NM groups, respectively. (C) Western blotting confirmed that ALDH3A1, keratocan and lumican were significantly more expressed in the RIFA medium + pCSE group than in the NM group. Compared with the RIFA medium group, ALDH3A1 was also more expressed in the RIFA medium + pCSE group. Fibronectin and α‐SMA were less expressed in the RIFA medium + pCSE group than the NM group (*P < .05; **P < .01; ***P < .001; ****P < .0001)

Immunofluorescence staining, Western blotting and qPCR were used to study the morphology and phenotype of the adherent hCSCs at P4 in RIFA medium, RIFA medium supplemented with pCSE and NM. After attaching for 24 h, the hCSCs at P1‐P4 in the RIFA medium supplemented with pCSE showed more physiological morphology. The majority of the adherent hCSCs had dendritic or stellate shapes and formed cellular networks with cell processes extending to connect with neighbouring cells as observed using light microscopy (Figure 2A). The hCSCs expressed keratocyte markers (ALDH3A1 and lumican) and were weakly stained with fibroblast and myofibroblast markers (fibronectin and α‐SMA) via immunofluorescence staining (Figure 2B). Additionally, many hCSCs at P3 were obviously positive to ALDH3A1 staining (Fig. S1). In contrast, the hCSCs at P1‐P4 in the NM had spindle shapes (Figure 2A) and were negligibly expressed ALDH3A1 but were strongly positive for lumican, fibronectin, and α‐SMA (Figure 2B). RT‐qPCR analysis showed that keratocyte marker related genes, such as *ALDH3A1*, *CD34*, *KERA* and *LUM*, were significantly greater in the RIFA medium supplemented with pCSE group than those in the NM group (14.08‐, 236.1‐, 79.1‐ and 12.57‐fold, respectively; *P* < .05). *ALDH3A1* and *CD34* were also more up‐regulated in the RIFA medium supplemented with pCSE group than in the RIFA medium group (8.2‐ and 13.54‐fold, respectively; *P* < .05) (Figure 3A). The fibrotic genes (*FN1* and *THBS1*) were significantly lower in the RIFA medium supplemented with pCSE group than in the NM group (0.26‐ and 0.32‐fold, respectively; *P* < .01). Other fibrotic genes (*COL3A1* and *ACTA2*) were significantly higher in the RIFA medium group than in the RIFA medium supplemented with pCSE (4.60‐fold; *P* < .01) and NM groups (5.94‐fold; *P* < .05) (Figure 3B). Western blotting confirmed that ALDH3A1, keratocan and lumican were significantly more expressed (7.19‐, 2.16‐ and 1.66‐fold, respectively; *P* < .05) in the RIFA medium supplemented with pCSE group. Compared with the RIFA medium group, ALDH3A1 was also more expressed in the RIFA medium supplemented with pCSE group (3.52‐fold; *P* < .05). Fibronectin and α‐SMA were less expressed (0.15‐ and 0.14‐folds, respectively; *P* < .01) in the RIFA medium supplemented with pCSE group than in the NM group (Figure 3C). Taken together, RIFA medium supplemented with 5 μg/ml pCSE can help hCSCs maintain the keratocyte phenotype and prevent hCSC fibrotic transition at least within P4 in vitro subcultures.

### 3.4 Improving hCSC proliferation and anti‐apoptosis using low‐serum RIFA medium supplemented with pCSE

**FIGURE 4:**
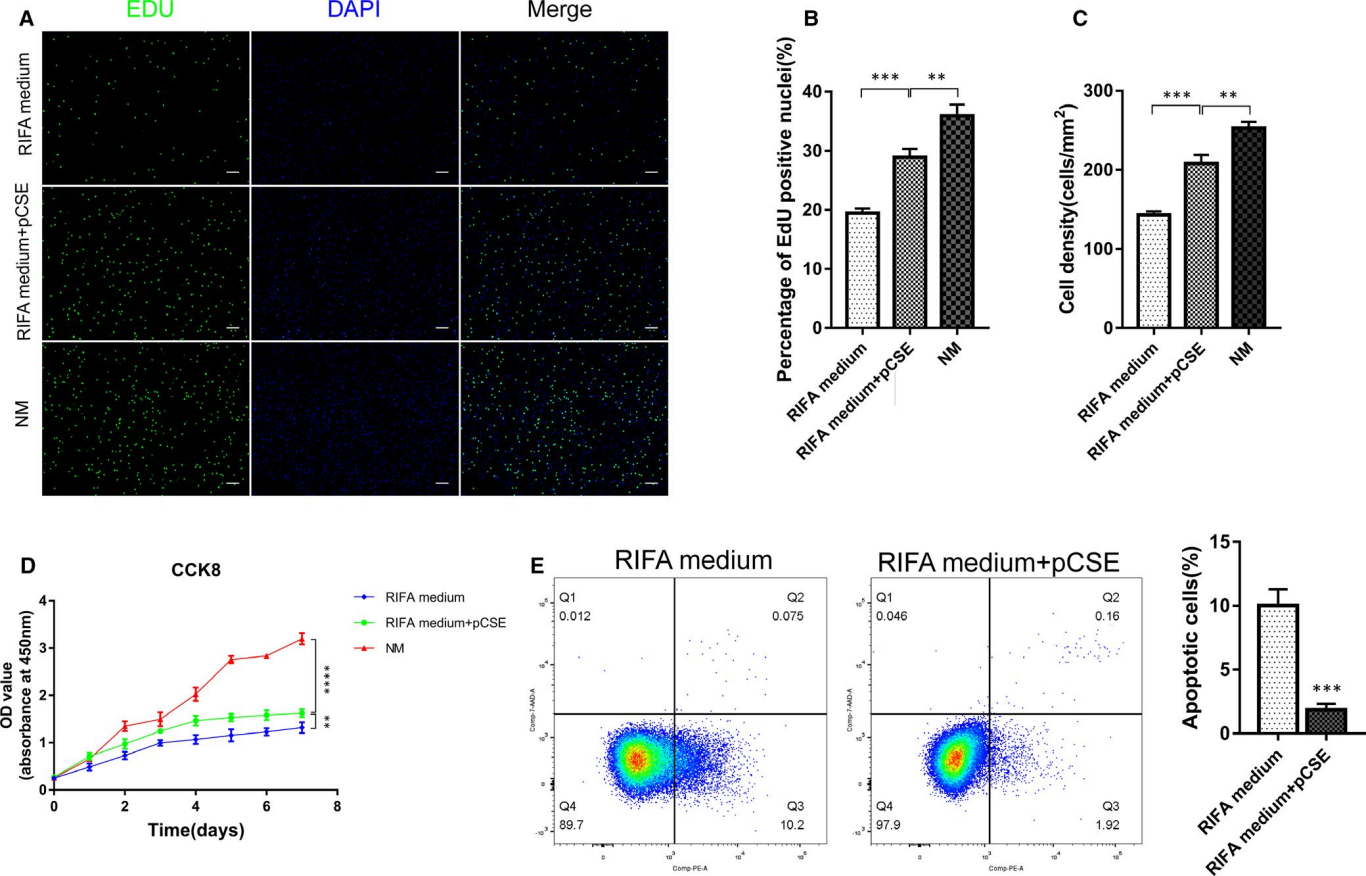
The effect on hCSC proliferation and apoptosis of RIFA medium + pCSE. (A‐C) The percentage of EdU‐positive cells and cell density in the RIFA medium group, RIFA medium + pCSE group and NM group. (D) The hCSC cytoactivity in the RIFA medium group, RIFA medium + pCSE group and NM group during 7 d via the CCK‐8 assay. (E) Compared to the RIFA medium group, RIFA medium + pCSE significantly reduced the apoptosis rate of the hCSCs (**P < .01; ***P < .001; ****P < .0001). Scale bars: 100 μm for A

To investigate the functions of pCSE in terms of hCSC proliferation, cytoactivity and anti‐apoptosis, EdU, CCK‐8 and H_2_O_2_‐induced apoptosis assays were conducted. After treatment with RIFA medium, RIFA medium supplemented with pCSE or NM for 2 d, the results of EdU assay demonstrated that, at the same seeding density, the hCSCs at P4 cultured in the NM had more EdU‐positive cells than those in the RIFA medium and RIFA medium supplemented with pCSE on day 2 (Figure 4A). The percentage of EdU‐positive hCSC nuclei in the RIFA medium, the RIFA medium supplemented with pCSE and the NM were 19.43 ± 0.47%, 28.88 ± 0.84% and 35.92 ± 1.08%, respectively (Figure 4B). There was a significant increase in the cell density in the RIFA medium supplemented with pCSE (208 ± 6.37 cells/mm^2^) compared with the RIFA medium (143.1 ± 2.53 cells/mm^2^) after 2 days of culture. The cell density in NM (253.1 ± 4.32 cells/mm^2^) was also significantly higher than the other two groups (Figure 4C). The CCK‐8 was used to assess cytoactivity of hCSCs cultured in different media, in which the results showed that the hCSC cytoactivity of NM group was significantly increased compared with other two groups, and that for RIFA medium supplemented with pCSE group was more prominent than in the RIFA medium group from day 2 (*P* < .01). The cell growth curve was further assessed for cytoactivity of hCSCs in different media (Figure 4D). Besides, following exposure to 500 μM H_2_O_2_ for 24 h, the apoptotic rate of the hCSCs in RIFA medium supplemented with pCSE (1.87 ± 0.27%) was lower than in the RIFA medium (10.03 ± 0.73%) (*P* < .01) (Figure 4E), indicating that the pCSE reduced the apoptosis of hCSCs when exposed to H_2_O_2_. Therefore, pCSE not only increases hCSC proliferation and cytoactivity, but also prevents hCSC apoptosis in vitro.

### 3.5 Proteomic identification of pCSE

We used the nano LC‐MS/MS assay and bioinformatic analysis to identify the proteomics of the pCSE. The nano LC‐MS/MS assay showed that there were approximately 740 kinds of pCSE proteins as listed in Table S2. The biological processes of the identified proteins were analysed by Metascape as represented by the gene symbols. Significant biological processes potentially affected by pCSE proteins were listed in Table 1, which included extracellular matrix organization, developmental growth, fibroblast proliferation and regulation of the apoptotic signalling pathway. The 10 most notable biological processes were illustrated (Table S3), including exocytosis regulation, supramolecular fibre organization, extracellular structure organization, negative regulation of proteolysis, negative regulation of endopeptidase activity and wound healing. Fig. S2A showed the significant biological processes of the identified pCSE specimen proteins and the protein count of each biological process. The 10 most notable biological processes in GO enrichment and the protein count of each biological process demonstrated the pCSE’s characteristics (Fig. S2B). The ELISA assay verified the concentration of decorin (90.65 ± 14.1 ng/ml), insulin‐like growth factor 2 (84.56 ± 11.7 ng/ml) and clusterin (196 ± 46.61ng/ml) (Fig. S3). In summary, the pCSE with its anti‐fibrosis, pro‐proliferation and anti‐apoptosis activities, is beneficial for hCSC cultivation.

**TABLE 1:** Significant biological processes potentially affected by porcine corneal stromal extract (pCSE) proteins using Metascape analysis

Protein functions	Significant biologican processes	LogP values	Gene symbols identified proteins
ECM‐related	Extracellular matrix organization	−39.485	A2M, AEBP1, AGT, ANXA2, BGN, SERPINH1, COL1A1, COL5A1, COL5A2, COL6A2, COL6A3, COL11A1, COL17A1, COMP, CTSL, DCN, FBLN1, FGA, FGG, FMOD, FN1, HSPG2, LOX, LUM, NID1, PLG, SERPINF2, SPARC, TGFBI, THBS1, TIMP1, COL14A1, VTN, FBLN5, ABI3BP, EFEMP2, COL18A1
Proliferation‐related	Developmental growth	−8.12379	AGT, ANXA1, ANXA2, APOD, APOE, CD81, COL6A2, COL6A3, COMP, DIO3, DPYSL2, ECM1, ENO3, FN1, G6PD, GDI1, GPX1, GSN, HSP90AA1, HSP90AB1, IGF2, PAFAH1B1, PKM, PPIB, PSAP, RBP4, SFRP1, SOD1, STC1, COL14A1, EZR, EIF4H, YBX3, STC2, IQGAP1, OLFM1, PDLIM5, DBNL, MTPN, PI16
	Fibroblast proliferation	−4.17899	AGT, ANXA2, AQP1, FN1, GSTP1, MIF, S100A6, SFRP1
	Positive regulation of fibroblast proliferation	−3.99958	AGT, ANXA2, AQP1, FN1, MIF, S100A6
	Positive regulation of growth	−3.61862	RHOA, CDC42, DIO3, F2, FN1, GDI1, IGF2, PAFAH1B1, PPIB, S100A8, SFRP1, EZR, YBX3, DBNL, MTPN
Apoptosis‐related	Regulation of apoptotic signaling pathway	−13.0717	AGT, CD44, CLU, ENO1, FGA, FGG, GPX1, PDIA3, GSN, GSTP1, HNRNPK, HSPA1B, HSPB1, LGALS3, LMNA, MIF, NMT1, P4HB, RPL11, RPS3, RPS7, S100A8, SFRP1, SOD1, PRDX2, THBS1, TPT1, YWHAB, YWHAE, YWHAG, YWHAH, YWHAZ, YBX3, PEA15, PDCD5, SLC9A3R1, RACK1, ERP29, YWHAQ, PARK7
	Apoptotic signaling pathway	−11.7243	AGT, CD44, CLU, ENO1, FGA, FGG, GPX1, PDIA3, GSN, GSTP1, HINT1, HNRNPK, HSPA1B, HSPB1, KRT18, LCN2, LGALS3, LMNA, MIF, NMT1, P4HB, RPL11, RPS3, RPS7, S100A8, SCN2A, SFRP1, SOD1, PRDX2, THBS1, TPT1, YWHAB, YWHAE, YWHAG, YWHAH, YWHAZ, YBX3, PEA15, PDCD5, SLC9A3R1, RACK1, ERP29, YWHAQ, PARK7
	Negative regulation of apoptotic signaling pathway	−6.30229	CD44, CLU, ENO1, FGA, FGG, GPX1, GSTP1, HSPA1B, HSPB1, LGALS3, LMNA, MIF, PRDX2, THBS1, TPT1, YBX3, PEA15, PARK7
	Negative regulation of intrinsic apoptotic signaling pathway	−4.41757	CD44, CLU, ENO1, GPX1, HSPB1, MIF, TPT1, YBX3, PARK7

### 3.6 Intrastromal injection of hCSCs into normal mouse corneas

**FIGURE 5:**
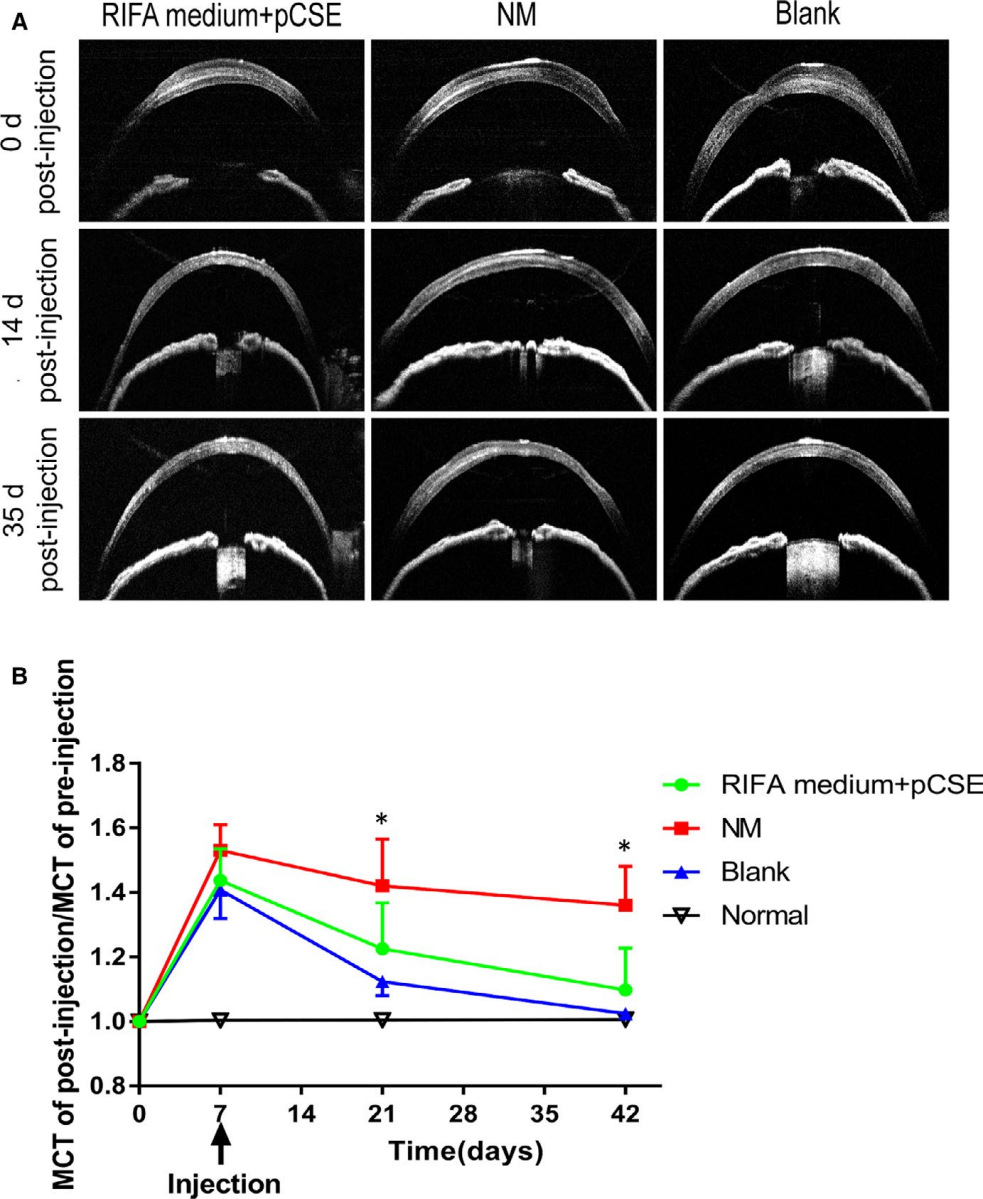
The changes in the mouse mean corneal thickness (MCT) before and after intrastromal injection. MCT measured by AS‐OCT imaging demonstrated a transient thickening of all of the corneas after injection. Partial high‐density images were found in all of the corneal stromal layers. The ratio between the post‐injection and pre‐injection MCT in the RIFA medium + pCSE group and blank group corneas gradually returned to normal levels while the NM group corneas remained thicker (*P < .05)

**FIGURE 6:**
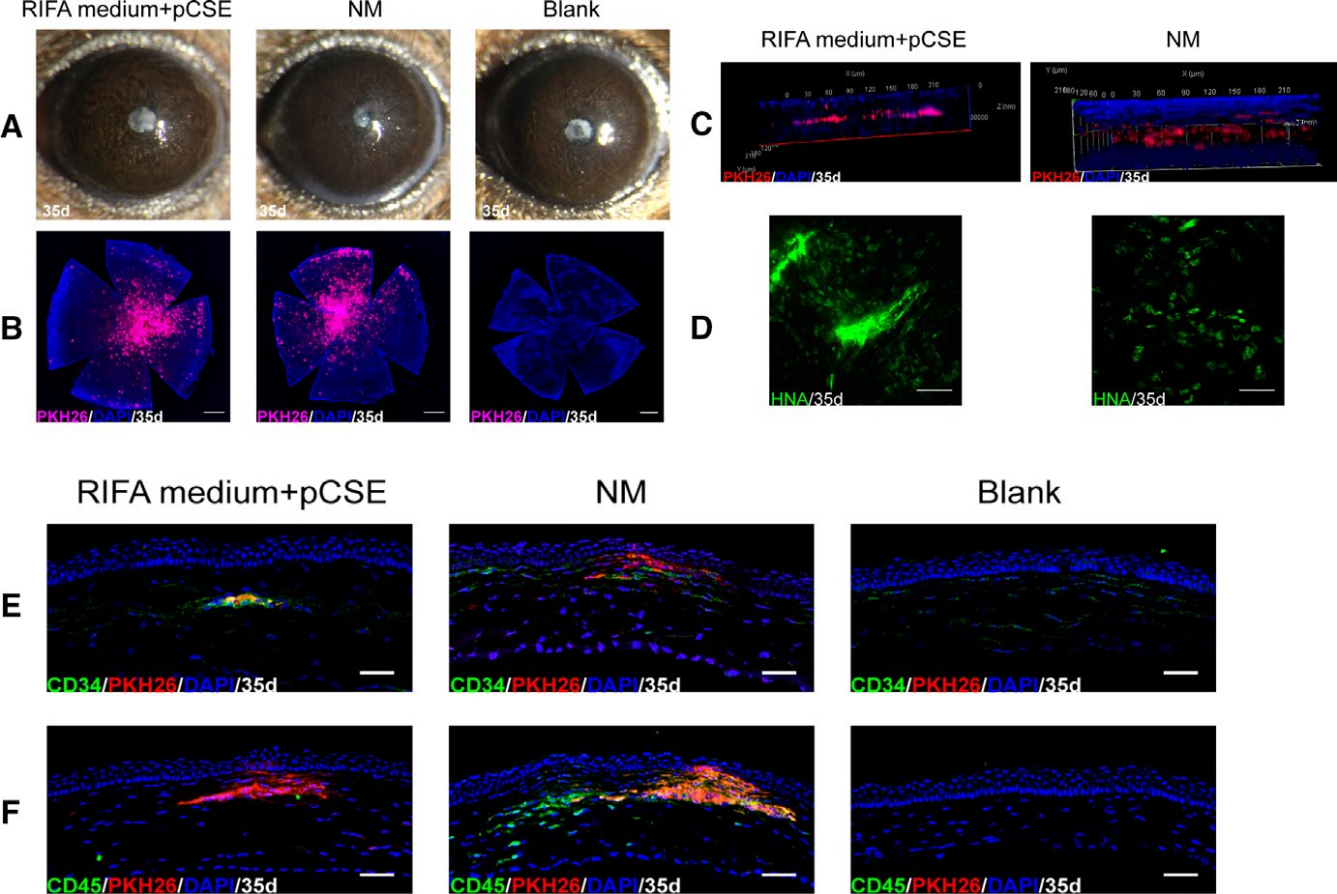
Mouse corneal changed by 35 days after injection. (A) Slit‐lamp biomicroscopy showed that the RIFA medium + pCSE group and the blank group corneas appeared to be transparent. In contrast, the NM group displayed visible haze. (B) Corneal whole‐mount staining illustrated persistence of the PKH26‐labelled hCSCs (red) in the central corneal region and few PKH26‐labelled hCSCs distributed in the corneal periphery. (C) A confocal microscopic image of the partially thick corneal tissue showed that the hCSCs in the NM group tended to vertically migrate, while the hCSCs in the RIFA medium + pCSE group seldom migrated in the z‐position. (D) Corneal whole‐mount staining showed that human nuclear antigen (HNA)‐positive cells were observed in both the RIFA medium + pCSE and NM group corneas. (E) The expression of human CD34 was positive in the RIFA medium + pCSE group and negligible in the NM and blank groups. (F) The CD45^+^ cells were localized around the injected hCSCs in the NM group corneas and negligible in the RIFA medium + pCSE and blank groups. Nuclei were stained with DAPI (blue). Scale bars: 500 μm for B; 50 μm for D, E and F

The injected corneas were divided into three groups: a RIFA medium supplemented with pCSE group, an NM group and a blank group (only DMEM/F12 without cells). After separate subculturing in RIFA medium supplemented with pCSE and NM in vitro, hCSCs at P4 were injected into mouse corneas to evaluate the hCSCs in vivo. To investigate whether the injected cells effected the corneal thickness, we assessed the mouse MCT using anterior segment AS‐OCT. The mouse corneas thickened post‐injection (Figure 5A). The corneal thickness ratio assessed via the MCT between pre‐injection and post‐injection had no significant difference in all of the groups before day 14. By day 14 after injection, the mouse corneal thickness gradually decreased. The corneal thickness ratio between pre‐injection and post‐injection in the NM group was significantly higher than in the RIFA medium supplemented with pCSE group and the blank group by days 14 and 35 after injection (*P* < .05) (Figure 5B). By day 35 after injection, the RIFA medium supplemented with pCSE group and the blank group appeared to be transparent, while the NM group demonstrated visible haze in the operated corneas (Figure 6A). In addition, corneal whole‐mount staining showed persistence of the PKH26‐labelled hCSCs (red) in the central corneal region. A few PKH26‐labelled hCSCs were distributed in the corneal periphery. Compared to the RIFA medium supplemented with pCSE group, gradual cell migration to the corneal periphery was obvious in the NM group (Figure 6B). Confocal microscopic imaging of the partially thick corneal tissue showed that the hCSCs in the NM group also tended to vertically migrate, while the hCSCs in the RIFA medium supplemented with pCSE seldom migrated in the z‐position (Figure 6C). Human nuclear antigen (HNA)‐positive cells were observed both in the RIFA medium supplemented with pCSE and the NM group corneas using corneal whole‐mount staining (Figure 6D), indicating that mouse corneal transparency was not significantly altered by the long‐term presence of hCSCs in the RIFA medium supplemented with pCSE.

By day 14 after injection, both the RIFA medium group and RIFA medium supplemented with pCSE group recovered to be transparent (Fig. S4A). Immunostaining of the mouse cornea sections showed that positive CD34 and negative CD45 both in the RIFA medium group and RIFA medium supplemented with pCSE group (Fig. S4B). By day 35 after injection, immunostaining demonstrated that the expression of human CD34 was positive in the RIFA medium supplemented with pCSE group and negligible in the NM and blank groups (Figure 6E). We also observed positive CD45 expression cells localized around the injected hCSCs in the NM group corneas and negligible in the RIFA medium supplemented with pCSE and blank groups (Figure 6F). Collectively, these results demonstrate the feasibility of intrastromal injection of hCSCs in the RIFA medium supplemented with pCSE, which did not increase the mouse MCT 35 days after injection. Similar to native keratocytes, these cells increase the expression of the appropriate markers without causing corneal haze and host inflammatory response in vivo.

## 4 DISCUSSION

There are presently several sources of hCSCs, including somatic CSCs from donated human corneal tissue, human corneal stromal stem cells (hCSSCs), mesenchymal stem cells, dental pulp stem cells and induced pluripotent stem cells (iPSCs).^14^, ^28^, ^29^ In the current study, we used fresh SMILE‐derived lenticules as new CSC sources of primary cells harvested from corneal central stroma for cell‐based therapy. Compared to the conventional approach, SMILE‐derived lenticules have some unique advantages. For example, many lenticules are available due to increasing numbers of young and healthy patients undergoing SMILE. Additionally, lenticules originate only from the human corneal stromal layer. This source ensures that pure native hCSCs can be harvested while avoiding contamination by corneal epithelial and endothelial cells. It was recently reported that no apparent and significant morphological or phenotype differences were observed between human CSCs produced from different locations (centre vs periphery) and isolation techniques (explant vs enzymatic digestion).^30^ Thus, we believe that SMILE‐derived lenticules are reliable sources of hCSCs. To the best of our knowledge, research on hCSC isolation and propagation for cell therapy from SMILE‐derived lenticule has not previously been reported.

Improving the quantity and quality of isolated cells from SMILE‐derived lenticules of central stroma rather than the peripheral region is an important issue. In this study, we found that the hCSCs in the RIFA medium supplemented with pCSE maintained the distinctive dendritic morphology and quiescent keratocyte phenotype of ALDH3A1 and lumican. Although a relatively small amount hCSCs at P4 kept positive to ALDH3A1, cells revealed negative to fibronectin and α‐SMA. Furthermore, many hCSCs at P3 were obviously positive to ALDH3A1 staining. Their proliferation improved after culturing and subculturing even at passage 4. However, the hCSCs at P4 in the NM had heterogeneity and activated fibroblast characteristics. The RIFA components included ROCK inhibitor Y27632, ITS, FGF2 and L‐ascorbate 2‐phosphate. Previous studies reported that Y‐27632 not only inhibited keratocyte‐to‐myofibroblast transitions in vitro,^31^, ^32^ but also promoted cell adhesion, survival and proliferation.^2^ ITS was regarded as an essential substitute for serum, promoting cellular growth and proliferation.^33^ A recent study suggested that ITS partially induced a quiescent keratocyte phenotype in vitro hCSC cultures.^34^ Studies demonstrated that FGF2 promoted cell proliferation and migration. However, there are contrary reports regarding FGF2’s effects. Some studies showed that FGF2 reduced the expression of α‐SMA and enhanced expression of KSPGs (keratocan and lumican).^35^, ^36^ However, other studies suggested that FGF2 was responsible for the down‐regulation or loss of the expression of KSPG in activated keratocytes. Chen et al reported that Rho/ROCK inhibition suppressed FGF2‐mediated loss in the expression of keratocan and lumican via the Jun N‐terminal kinase (JNK) signalling pathway.^31^, ^37^ L‐ascorbate 2‐phosphate stimulated CSCs to synthesize the ECM, mimicking its functions in native corneas. Funderburgh and colleagues reported that they utilized keratocyte differentiation medium (KDM), which consisted of Advanced DMEM, AlbuMAX II, insulin recombinant full chain, L‐ascorbic acid‐2‐phosphate, L‐alanyl‐L‐glutamine, FGF2 and TGF‐β3, to induce the differentiation of hCSSCs into keratocytes. After cultured in KDM, hCSSCs differentiated into keratocytes with significant up‐regulation of the keratocyte gene markers, including *KERA*, *B3GnT7* and *CHST6*.^38^ Additionally, Scott el al. used serum‐free keratocyte DMEM/F12 growth medium with ITS, FGF2 and phospho‐ascorbic acid to successfully maintain dendritic morphology in human keratocytes.^39^ Our study was consistent with the reported findings without down‐regulating the expression of KSPGs in the propagated hCSCs.

Characterizing the ECM, which is the backbone of the cornea, is key for elucidating the differences between corneal stroma cells in vivo and ex vivo.^30^ Our proteomic results using nano LC‐MS/MS and Metascape analysis demonstrated that there were several significant biological processes potentially affected by pCSE in GO enrichment, such as extracellular matrix organization, fibroblast proliferation and apoptotic signalling pathway, though some of these biological processes were not at the top significance. Therefore, we presume that the cross‐talk of proteins in pCSE with hCSCs participates in a range of biological processes, including anti‐fibrosis transition, growth or proliferation promotion and apoptosis inhibition, which are beneficial for hCSC culture.

Recently, Tseng and coworkers revealed that HC‐HA/PTX3, a high molecular weight hyaluronic acid (HA) covalently linked with heavy chain 1 (HC1) from inter‐α‐trypsin inhibitor and further complexed with pentraxin 3 (PTX3), purified from human amniotic membrane could revert human corneal fibroblasts and myofibroblasts to keratocytes by inhibiting canonical TGF‐β signalling and by activating BMP signalling, which exerted an antiscarring action.^40^ In our study, we supplemented pCSE to propagate CSCs. Compared with AME, pCSE has many unique advantages. First, as natural and niche ECM components for native CSC survival in vivo, pCSE has a large array of biomolecules and proteins beneficial for CSC growth and expansion. Nagymihály et al reported that cultivated CSCs underwent a complete surface marker and genotype profile change compared with CSCs in situ. The gene expression and protein profile of native CSCs compared with cultured CSCs in vitro showed that the latter likely adapted from an in vivo ECM niche to an adopted environment in the presence of serum in the culture medium.^30^ Thus, supplementing pCSE with ECM niche component in culture medium provides a more favourable environment for subculturing hCSCs in vitro. In addition, fresh porcine corneas are widely available and pCSE can be easily prepared using this tissue. Porcine corneal tissue is also adaptable to hCSCs. Porcine corneas have been successfully used for corneal xenotransplantation over the past decade.^41^ Li et al^42^ reported that keratocytes started to repopulate from human recipient tissue into the acellular porcine corneal stroma between 3 and 6 months post‐operatively via proliferation and migration. The results clinically indicated that ECM components of porcine corneas encourage human keratocyte ingrowth and demonstrated good biological stability.

Intrastromal injection is usually considered a simple, feasible, therapeutic and minimally invasive procedure to deliver hCSCs to the cornea.^43^ This study also demonstrated the feasibility of intrastromal injection of adult hCSCs in vivo. Transient corneal opacity was generated by the injection of cell suspension, which disappeared within hours to days. However, the corneas in the RIFA medium group and RIFA medium supplemented with pCSE group were transparent, while the NM group displayed haze in the operated cornea. In the RIFA medium supplemented with pCSE group, corneal whole mounting also demonstrated the persistence of the PKH26‐labelled hCSCs in the central cornea with few PKH26‐labelled hCSCs in the corneal periphery. However, in the NM group, more injected cells migrated to the corneal periphery. Previous reports demonstrated that injected cells initially remained at the injection site and gradually migrated to the corneal periphery or closer to the peripheral stroma.^43^, ^44^ This uncontrolled migration could limit intrastromal injection if cells are required in a target area where they can fulfil their therapeutic function.^43^ The horizontal and vertical migration of injected cells was relevant and consistent with our corneal thickness MCT results between pre‐injection and post‐injection, which demonstrated that the ratio in the NM group was significantly higher than in the RIFA medium supplemented with pCSE group by day 35 after injection. Thus, we postulate that the injected hCSCs in the NM group were activated and more likely to migrate, which might have increased the corneal thickness. CD34^+^ expressing cells represent quiescent keratocytes, whereas CD34^‐^ cells are activated stromal cells and stromal fibroblasts do not fully revert to the quiescent condition.^45^ Yam et al^46^ reported that post‐natal periodontal ligament cells injected into porcine corneas expressed CD34 after corneal organ culture for 7 days. In our study, CD34 exhibited negative expression in vitro (data not shown) but positive in vivo, indicating that the injected cells transitioned to the quiescent condition in the native physiologic condition.

We obtained a substantial amount of hCSCs via significant serum supplementation (5%‐10%). However, after intrastromal injection, these fibroblast characteristic cells did not produce KSPGs and stromal crystallins but induced corneal haze, host inflammatory response and corneal neovascularization (CNV).^43^ Prior research reported that the expression of CD45 (a leucocyte marker) was negligible in corneal stromal keratocyte‐injected rat corneas, even in fibroblast‐injected corneas, at 2 and 4 weeks post‐injection.^43^ In contrast, we did not find CD45^+^ cells in the corneas in the RIFA medium supplemented with pCSE group but they were present in the NM group corneas by day 35 after injection. CNV was negligible in all of the operated corneas. These results indicated there were no obvious adverse reactions after the intrastromal injection of hCSCs in the low‐serum RIFA medium supplemented with pCSE.

The limitation of this study is that hCSCs were injected into normal mouse corneal stroma rather than the corneal injury model. And the short‐term animal experiments between RIFA medium and RIFA medium supplemented with pCSE group were not obviously different. The long‐term animal studies will be our future work. Additionally, according to our nano LC‐MS/MS results, there were approximately 740 proteins involved in pCSE. Thus, further research into the links and mechanisms by which proteins and CSCs propagate is needed. In the future, we will devote more time to observing injected hCSC interactions with cornea‐resident cells. These are crucial factors for clinical applications.

In conclusion, this study on the potential of hCSCs suggested that soluble pCSE with low‐serum RIFA medium improved the proliferation of hCSCs from SMILE‐derived lenticules with the properties of physiological keratocytes. The pCSE with its anti‐fibrosis, pro‐proliferation, and anti‐apoptosis activities, was beneficial for hCSC cultivation. The intrastromal injection of hCSCs into normal mouse corneas demonstrated that the hCSCs in the low‐serum RIFA medium supplemented with pCSE led to a more rapid recovery of corneal thickness and transparency, less migration, positive CD34 expression and negative CD45 expression in corneas compared with implanted hCSCs cultured in the NM. Therefore, using pCSE with low‐serum RIFA medium not only promoted hCSC proliferation, but also maintained the physiological function of human keratocytes. The hCSCs from the SMILE‐derived lenticules in the pCSE with low‐serum RIFA medium demonstrated good potential for corneal stromal cell therapy.

## CONFLICT OF INTEREST

The authors declare no conflict of interest.

## AUTHOR CONTRIBUTIONS

Shenyang Li: Research performance and manuscript writing. Shenyang Li, Zekai Cui, Jianing Gu and Yini Wang: Data analysis. Shenyang Li, Zekai Cui, Jiansu Chen and Shibo Tang: Design research study. Shenyang Li, Zekai Cui and Jiansu Chen: Participation in manuscript writing and manuscript correction. All authors read and approved the final manuscript.

## Supporting information

Fig S1

Fig S2

Fig S3

Fig S4

Table S1

Table S2

Table S3

Supplementary Material

Supplementary Material

Supplementary Material

Supplementary Material

Supplementary Material
