# Supplementary figures and images for: Effect of porcine corneal stromal extract on keratocytes from SMILE‐derived lenticules
Source: J Cell Mol Med. 2020 Dec 20;25(2):1207–20. doi: 10.1111/jcmm.16189 (PMC7812260; doi:10.1111/jcmm.16189)

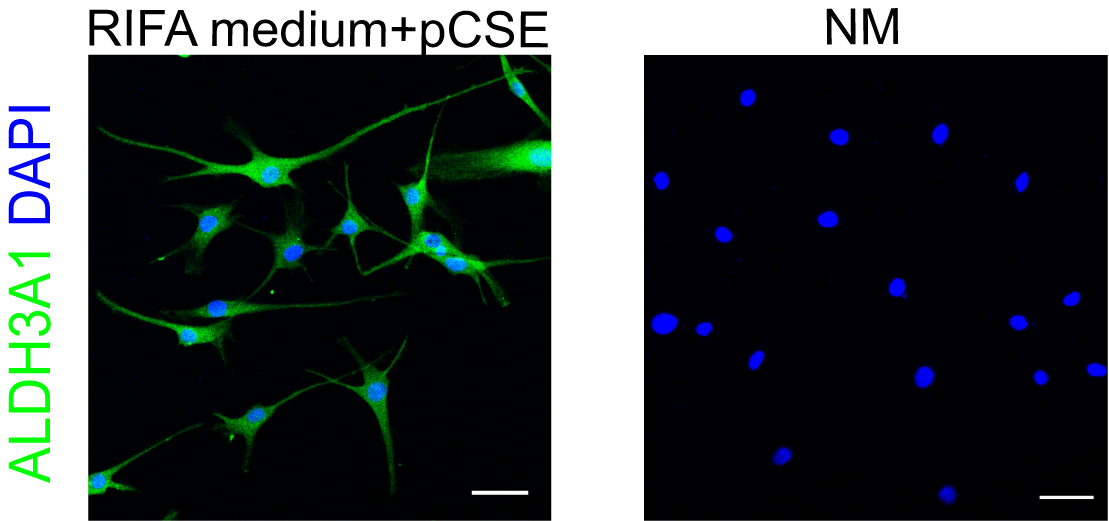

Supplement: Supplementary file 1 — Fig S1 [file JCMM-25-1207-s001.tif]

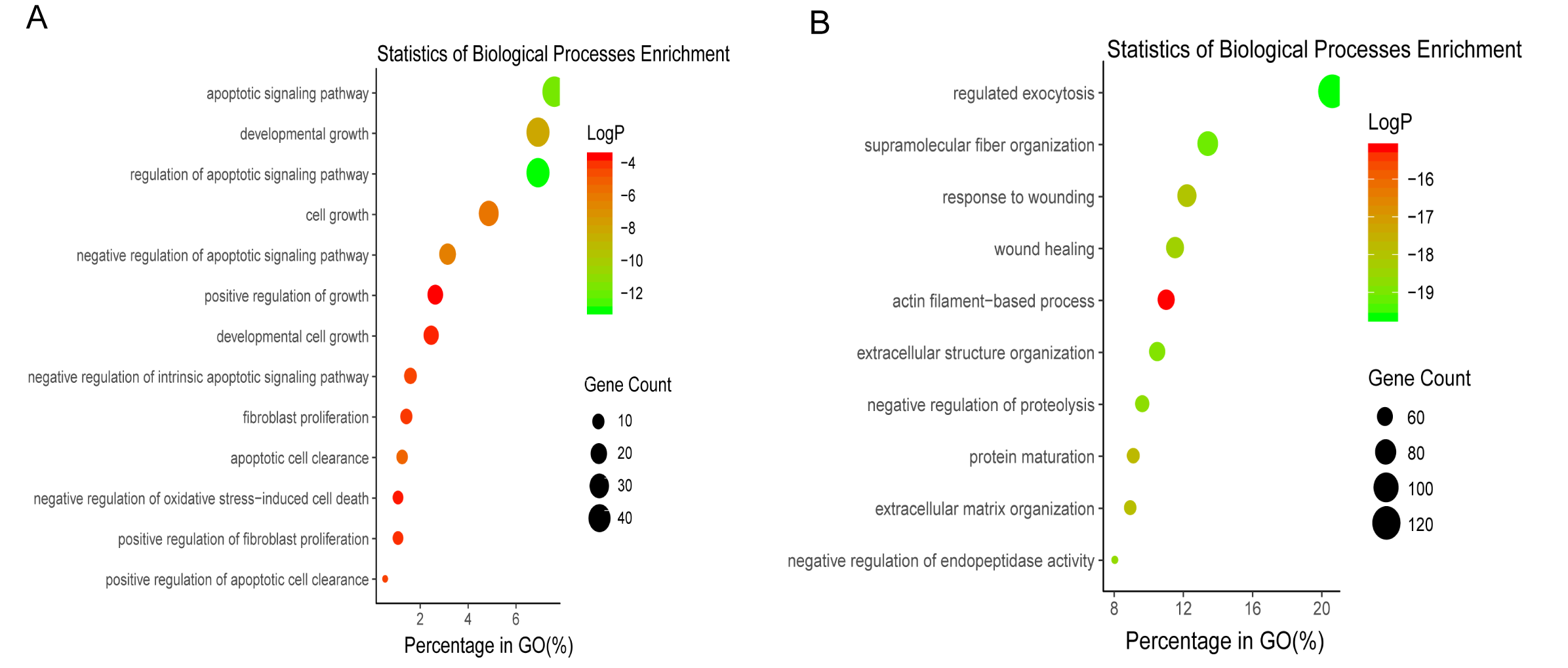

Supplement: Supplementary file 2 — Fig S2 [file JCMM-25-1207-s002.tif]

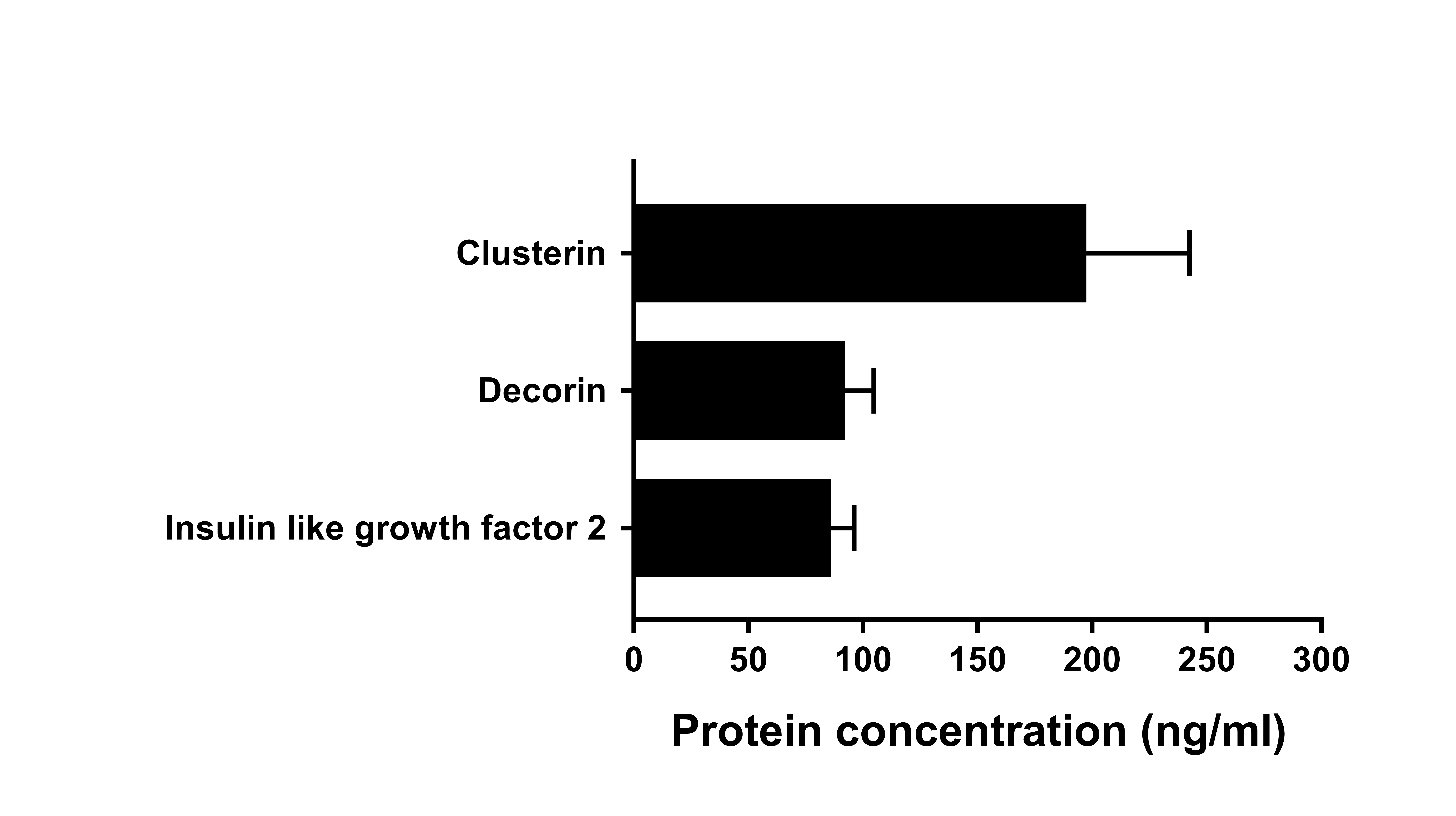

Supplement: Supplementary file 3 — Fig S3 [file JCMM-25-1207-s003.tif]

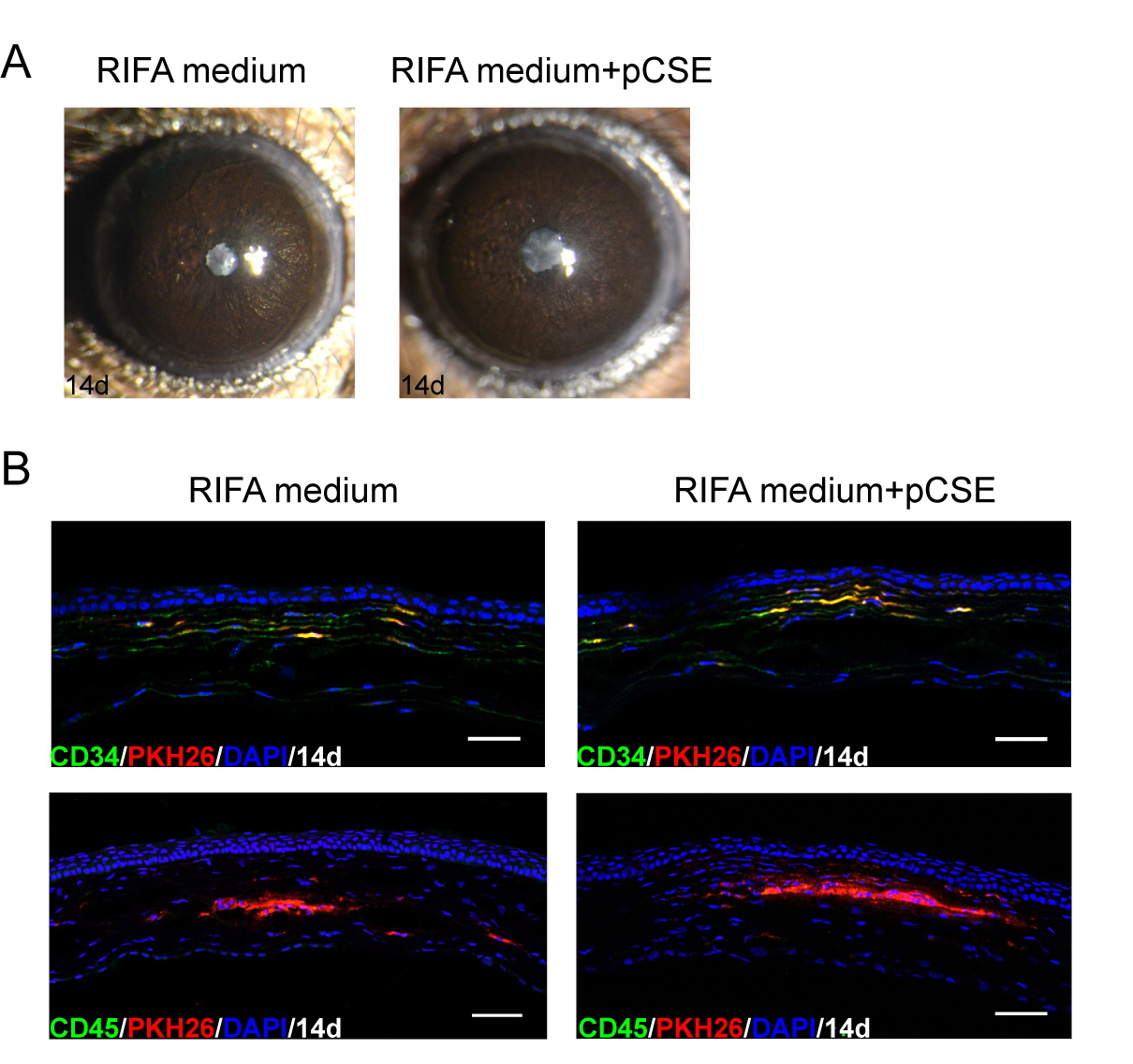

Supplement: Supplementary file 4 — Fig S4 [file JCMM-25-1207-s004.tif]
