# Supplementary material for: Effect of porcine corneal stromal extract on keratocytes from SMILE‐derived lenticules
Source: J Cell Mol Med. 2020 Dec 20;25(2):1207–20. doi: 10.1111/jcmm.16189 (PMC7812260; doi:10.1111/jcmm.16189)
Supplement: Supplementary file 5 — Table S1 [file JCMM-25-1207-s005.docx]

| **Gene** | **Forward Primer（5’—3’）** | **Reverse Primer（5’—3’）** | **Product size (bp)** | **GenBank accession ID** |
| --- | --- | --- | --- | --- |
| ALDH3A1 | TGTTCTCCAGCAACGACAAGG | AGGGCAGAGAGTGCAAGGT | 108 | NM_001330150.1 |
| CD34 | CTACAACACCTAGTACCCTTGGA | GGTGAACACTGTGCTGATTACA | 185 | NM_001773.2 |
| KERA | AACCTGACCCTTCTTGACCT | ACTGCATTGTATTGGCTGGT | 154 | NM_007035.3 |
| LUM | GCTTCAATCAGATAGCCAGAC | CAGCCAGTTCGTTGTGAGA | 153 | NM_002345.3 |
| COL3A1 | AAAGGCGAAGATGGCAAGGA | GTTCTCCAGCAGCTCCTCTG | 190 | [NM_000090.3](https://www.ncbi.nlm.nih.gov/nucleotide/NM_000090.3?report=genbank&log$=nucltop&blast_rank=1&RID=PMPKT2DC015) |
| FN1 | AAGACCATACCCGCCGAATG | GGCATTTGGATTGAGTCCCG | 109 | [NM_212482.2](https://www.ncbi.nlm.nih.gov/nucleotide/NM_212482.2?report=genbank&log$=nucltop&blast_rank=10&RID=YJ159959014) |
| THBS1 | TATAGCGACCCCATGTACCG | AGTCTTCCTGCCCTGAGTTG | 181 | [NM_003246.3](https://www.ncbi.nlm.nih.gov/nucleotide/NM_003246.3?report=genbank&log$=nucltop&blast_rank=2&RID=PMPRFNZR015) |
| ACTA2 | TATCCCCGGGACTAAGACGG | CACCATCACCCCCTGATGTC | 185 | NM_001141945.2 |
| GAPDH | GGAAGGTGAAGGTCGGAGTC | GATCTCGCTCCTGGAAGATGG | 240 | [NM_002046.6](https://www.ncbi.nlm.nih.gov/nucleotide/NM_002046.6?report=genbank&log$=nucltop&blast_rank=3&RID=YJ11N366016) |

Table S1 List of primers.
