# Supplementary material for: Effect of porcine corneal stromal extract on keratocytes from SMILE‐derived lenticules
Source: J Cell Mol Med. 2020 Dec 20;25(2):1207–20. doi: 10.1111/jcmm.16189 (PMC7812260; doi:10.1111/jcmm.16189)
Supplement: Supplementary file 7 — Table S3 [file JCMM-25-1207-s007.docx]

Table S3. The top 10 most notable biological processes in GO enrichment

| Most Notable Biological Processes | LogP Values | Gene Symbols Identified Proteins |
| --- | --- | --- |
| regulated exocytosis | -19.6571 | A1BG, A2M, ACTN4, AGL, AHSG, ALB, ALDOA, ALDOC, ANPEP  ANXA2, ANXA5, APOH, ARF1, RHOA, B2M, SERPING1, C3, CAPN1  CAT, CD44, CHI3L1, CLU, CST3, CSTB, CTSB, CTSD, CTSZ, CFD  DSG1, ECM1, EEF1A1, EEF2, SERPINB1, FABP5, FCN1, FGA, FGG  FLNA, FN1, GDI2, GPI, GSN, GSTP1, HBB, HMGB1, HP, HRG, HSPA1B  HSPA6, HSP90AA1, HSP90AB1, IDH1, IGF2, ITIH4, KNG1, KPNB1  KRT1, LCN2, LGALS3, LGALS3BP, LTA4H, LTF, MIF, NME2, PNP  ORM1, CFP, PGAM1, PGM1, SERPINA1, PKM, PLG, SERPINF2, PPIA  PROS1, PSAP, QSOX1, PYGB, PYGL, RAB6A, RAC1, RAP1A, RARRES2, S100A8, S100A11, S100A12, S100A13, SOD1, SPARC, SPTAN1, TF, THBS1, TIMP1, TLN1, TMSB4X, CLEC3B, TTR, VCP, RAB7A, TAGLN2, IQGAP1, MGAM, PRDX6, WDR1, ACTR2, TUBB4B, PRDX4, CCT2, COTL1, QPCT, OSTF1, DBNL, DPP7, SDF4, CPPED1, NIT2, NAPRT, LRG1, CD109, TUBB |
| supramolecular fiber organization | -19.1181 | ACTA1, ACTG1, ACTN4, AEBP1, ALDOA, BIN1, ANXA2, APOE, ARF1, RHOA, B2M, CAPG, CAPZB, SERPINH1, CDC42, CFL1, CFL2, CLU, COL1A1, COL5A1, COL5A2, COL11A1, COMP, CRYAB, CSRP1, CST3, DPYSL3, FKBP1A, FLNA, FMOD, GPX1, GSN, HSPA1B, HSP90AB1, KPNB1, KRT2, KRT14, LOX, LUM, MARCKS, PAFAH1B1, PFN1, SERPINF2, PLOD1, PLS3, RAC1, RDX, RPS3, S100A10, SFRP1, FSCN1, SPTAN1, SPTBN1, TMSB4X, TPM1, TPM2, TPM3, TPM4, HSP90B1, COL14A1, EZR, VIM, TMSB10, WDR1, ARPC4, ARPC3, ACTR3, ACTR2, FBLN5, DSTN, PARK7, COTL1, CORO1C, EML2, DBNL, EFEMP2, TPPP3, MTPN |
| extracellular structure organization | -18.8537 | A2M, AEBP1, AGT, ALB, ANXA2, APOA2, APOA4, APOC3, APOE, ARF1, BGN, CAPG, CAPN1, CAPNS1, SERPINH1, CD44, CDH1, COL1A1, COL5A1, COL5A2, COL6A2, COL6A3, COL11A1, COL17A1, COMP, CPB2, CST3, CTSL, DCN, FBLN1, FGA, FGG, FMOD, FN1, GSN, HSPG2, KLKB1, LOX, LUM, NID1, P4HB, SERPINB5, PLG, SERPINF2, PLOD1, PLTP, QSOX1, SDC1, FSCN1, SPARC, TGFBI, THBS1, TIMP1, TTR, COL14A1, VTN, FBLN5, PRDX4, ABI3BP, EFEMP2, COL18A1 |
| negative regulation of proteolysis | -18.7123 | A2M, AGT, AHSG, AMBP, BIN1, AQP1, SERPINC1, SERPING1, C3, C4A, C4BPA, C5, CAST, SERPINH1, CD44, COL6A3, CPB2, CRYAB, CST3, CSTB, CTSZ, ECM1, SERPINB1, F2, GAPDH, GPX1, SERPIND1, HRG, HSP90AB1, ITIH1, ITIH4, KNG1, LTF, PEBP1, SERPINF1, SERPINA1, SERPINB5, SERPINB9, SERPINF2, PROS1, PZP, RENBP, RPL11, RPS7, SERPINA7, THBS1, TIMP1, VTN, YWHAE, PRDX3, PARK7, PRDX5, CD109, PI16, HMSD, ANXA8 |
| negative regulation of endopeptidase activity | -18.6076 | A2M, AGT, AHSG, AMBP, BIN1, AQP1, SERPINC1, SERPING1, C3, C4A, C5, CAST, SERPINH1, CD44, COL6A3, CRYAB, CST3, CSTB, SERPINB1, GAPDH, GPX1, SERPIND1, HRG, ITIH1, ITIH4, KNG1, LTF, PEBP1, SERPINF1, SERPINA1, SERPINB5, SERPINB9, SERPINF2, PROS1, PZP, RENBP, SERPINA7, THBS1, TIMP1, VTN, YWHAE, PRDX3, PARK7, PRDX5, CD109, HMSD, ANXA8 |
| wound healing | -18.3753 | A2M, ANXA1, ANXA2, ANXA5, APOE, APOH, SERPINC1, SERPING1, COL1A1, COL5A1, COMP, DCN, F2, F9, F12, FBLN1, FGA, FGG, FN1, HRG, KNG1, KRT1, LOX, SERPINA1, PKM, PLG, SERPINF2, S100A8, SPARC, THBS1, TIMP1, VTN, ANXA8 |
| response to wounding | -18.0249 | A2M, ACTB, ACTG1, ANXA1, ANXA2, ANXA5, APOD, APOE, APOH, RHOA, SERPINC1, SERPING1, CAPZB, CD44, CD81, CDC42, CLIC1, COL1A1, COL5A1, COMP, CPB2, CSRP1, DCN, DPYSL3, ENO3, F2, F9, F10, F12, FBLN1, FGA, FGG, FLNA, FN1, FOLR1, GPX1, GSN, HBB, SERPIND1, HMGCR, HRG, HSPB1, KLKB1, KNG1, KRT1, KRT6A, LGALS1, LOX, SERPINA1, PKM, PLG, SERPINF2, PROC, PROS1, RAC1, S100A8, SDC1, SOD1, SPARC, PRDX2, THBS1, TIMP1, TLN1, TPM1, VTN, YWHAZ, MYL9, AHNAK2, CD109, MTPN, ANXA8 |
| extracellular matrix organization | -17.8695 | A2M, AEBP1, AGT, ANXA2, BGN, CAPG, CAPN1, CAPNS1, SERPINH1, CD44, CDH1, COL1A1, COL5A1, COL5A2, COL6A2, COL6A3, COL11A1, COL17A1, COMP, CPB2, CST3, CTSL, DCN, FBLN1, FGA, FGG, FMOD, FN1, GSN, HSPG2, KLKB1, LOX, LUM, NID1, SERPINB5, PLG, SERPINF2, PLOD1, QSOX1, FSCN1, SPARC, TGFBI, THBS1, TIMP1, TTR, COL14A1, VTN, FBLN5, PRDX4, ABI3BP, EFEMP2, COL18A1 |
| protein maturation | -17.7464 | A2M, AEBP1, ANXA2, APOH, CFB, SERPING1, C1QB, C1QC, C1R, C2, C3, C4A, C4BPA, C5, C6, C7, C8A, C8B, C8G, C9, CALR, CAST, SERPINH1, CD5L, CD81, CLU, COMP, CPB2, CTSZ, ENO1, F2, F9, F12, FGA, FGG, FKBP1A, GSN, CFH, HSPD1, CFI, IGKC, KLKB1, CFP, PGK1, SERPINF2, PROS1, THBS1, CLEC3B, VTN, SPON1, PRDX4, PISD, CPXM2 |
| actin filament-based process | -15.1724 | ACTA1, ACTB, ACTG1, ACTN4, ALDOA, BIN1, ANXA1, AQP1, ARF1, RHOA, ARHGDIA, ARHGDIB, CALR, CAPG, CAPZB, CDC42, CFL1, CFL2, CSRP1, DPYSL3, DSC2, EPB41L2, FLNA, GSN, HRG, MARCKS, MYL1, MYL6, PAFAH1B1, PFN1, SERPINF2, PLS3, RAC1, RDX, S100A10, SFRP1, FSCN1, SPTAN1, SPTBN1, STC1, TLN1, TMSB4X, TNNC2, TPM1, TPM2, TPM3, TPM4, HSP90B1, EZR, VIM, TMSB10, SLC9A3R1, WDR1, ARPC4, ARPC3, ACTR3, ACTR2, PDLIM5, DSTN, COTL1, CORO1C, DBNL, PHPT1, MTPN |
