# Supplementary material for: Effect of porcine corneal stromal extract on keratocytes from SMILE‐derived lenticules
Source: J Cell Mol Med. 2020 Dec 20;25(2):1207–20. doi: 10.1111/jcmm.16189 (PMC7812260; doi:10.1111/jcmm.16189)
Supplement: Supplementary file 8 — Supplementary Material [file JCMM-25-1207-s008.docx]

| **Analysis Report**   \| **Project Name** \| Label-free-based Quantitative Proteomic Analysis \| \| --- \| --- \|     **Analysis Report**  **1. Materials and Methods**  **1.1 Chemicals and Instrumentation**  Urea, Triethylammonium bicarbonate buffer (1.0 M, pH 8.5±0.1), iodoacetamide (IAA), formic acid (FA), acetonitrile (ACN), methanol, were purchased from Sigma (St. Louis, MO, USA). Trypsin from bovine pancreas was purchased from Promega (Madison, WI, USA). Ultrapure water was prepared from a Millipore purification system (Billerica, MA, USA). a Dionex Ultimate 3000 Nano LC system coupled with a Thermo Scientific Q Exactive (Thermo Fisher Scientific, USA) with an ESI nanospray source.  **1.2 Sample Preparation**  **1.2.2. Protein Digestion**   1. Transfer 100 μg protein into Microcon devices YM-10 (Millipore). The device was centrifuged at 12,000g at 4°C for 10 min. 2. 200 μL of 50 mM ammonium bicarbonate were added to the concentrate followed by centrifugation and repeat once. 3. After reduced by 10 mM DTT at 56°C for 1 h and alkylated by 20 mM IAA at room temperature in dark for 1h, the device was centrifuged at 12,000g at 4°C for 10 min and wash once with 50 mM ammonium bicarbonate. 4. Add 100 μL of 50 mM ammonium bicarbonate and free trypsin into the protein solution at a ratio of 1:50, and the solution was incubated at 37°C overnight. 5. The device was centrifuged at 12,000g at 4°C for 10 min. 100 μL of 50 mM ammonium bicarbonate was added into the device and centrifuged, and then repeat once. 6. Lyophilize the extracted peptides to near dryness. Resuspend peptides in 50 μL of 0.1% formic acid before LC-MS/MS analysis.   **1.3 Nano LC-MS/MS Analysis**  **1.3.1 nanoLC**  Nanoflow UPLC：Easy-nLC1000 (ThermoFisher Scientific, USA);  Nanocolumn：100 μm×10 cm in-house made column packed with a reversed-phase ReproSil-Pur C18-AQ resin (3 μm, 120 Å, Dr. Maisch GmbH, Germany);  Loaded sample volume: 5 μL  Mobile phase: A: 0.1% formic acid in water; B: 0.1% formic acid in acetonitrile.  Total flow rate : 300 nL/min  LC linear gradient: from 4% to 10% B for 5min, from 10% to 22% B for 80min, from 22% to 40% B for 25min. From 40% to 95% B for 5min and from 95% to 95% B for 5min.  **1.3.2 Mass spectrometry**  Thermo Scientific Q Exactive (Thermo Fisher Scientific, USA)  Resolution：70,000  AGC target：3e6  Maximum IT：40 ms  Scan range：350 to 1800 m/z  Resolution：17,500  AGC target：1e5  Maximum IT： 60 ms  TopN ：20  NCE / stepped NCE：27  **1.4 Data analysis**  The raw MS files were analyzed and searched against protein database based on the species of the samples using Maxquant (1.5.6.5). The parameters were set as follows: the protein modifications were carbamidomethylation (C) (fixed), oxidation (M) (variable); the enzyme specificity was set to trypsin; the maximum missed cleavages were set to 2; the precursor ion mass tolerance was set to 20 ppm, and MS/MS tolerance was 0.6 Da. Only high confident identified peptides were chosen for downstream protein identification analysis. |
| --- | --- | --- |
