# Supplementary material for: Effect of porcine corneal stromal extract on keratocytes from SMILE‐derived lenticules
Source: J Cell Mol Med. 2020 Dec 20;25(2):1207–20. doi: 10.1111/jcmm.16189 (PMC7812260; doi:10.1111/jcmm.16189)
Supplement: Supplementary file 11 — Supplementary Material [file JCMM-25-1207-s011.docx]

**FOR RESEARCH USE ONLY.**

**NOT FOR USE IN DIAGNOSTIC PROCEDURES.**

**Porcine Insulin-like growth factor 2 (IGF-2) ELISA Kit instruction**

**Intended use**

This IGF-2 ELISA kit is intended Laboratory for Research use only and is not for use in diagnostic or therapeutic procedures.The Stop Solution changes the color from blue to yellow and the intensity of the color is measured at 450 nm using a spectrophotometer. In order to measure the concentration of IGF-2 in the sample, this IGF-2 ELISA Kit includes a set of calibration standards. The calibration standards are assayed at the same time as the samples and allow the operator to produce a standard curve of Optical Density versus IGF-2 concentration. The concentration of IGF-2 in the samples is then determined by comparing the O.D. of the samples to the standard curve.

**Sample collection and storages**

**Serum** - Use a serum separator tube and allow samples to clot for 30 minutes before centrifugation for 10 minutes at approximately 3000×g. Remove serum and assay immediately or aliquot and store samples at -20℃ or -80℃.Avoid repeated freeze-thaw cycles

**Plasma** - Collect plasma using EDTA or heparin as an anticoagulant. Centrifuge samples for 30 minutes at 3000×g at 2-8℃ within 30 minutes of collection. Store samples at -20℃or -80℃. Avoid repeated freeze-thaw cycles.

**Cell culture supernates and other biological fluids -** Remove particulates by centrifugation and assay immediately or aliquot and store samples at -20℃or -80℃. Avoid repeated freeze-thaw cycles.

**
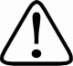
Note:** The samples shoule be centrifugated dequately and no hemolysis or granule was allowed.

**Materials required but not supplied**

1. Standard microplate reader(450nm)

2. Precision pipettes and Disposable pipette tips.

3. 37 ℃ incubator

**Precautions**

1. Do not substitute reagents from one kit to another. Standard, conjugate and microplates are matched for optimal performance. Use only the reagents supplied by manufacturer.

2. Do not remove microplate from the storage bag until needed. Unused strips should be stored at 2-8°C in their pouch with the desiccant provided.

3. Mix all reagents before using.

Remove all kit reagents from refrigerator and allow them to reach room temperature ( 20-25°C)

**Materials supplied**

| Name | 96 determinations | 48 determinations |
| --- | --- | --- |
| Microelisa stripplate | 12*8strips | 12*4strips |
| Standard | 0.3ml*6tubes | 0.3ml*6tubes |
| Sample Diluent | 6.0ml | 3.0ml |
| HRP-Conjugate reagent | 10.0ml | 5.0ml |
| 20X Wash solution | 25ml | 15ml |
| Chromogen Solution A | 6.0ml | 3.0ml |
| Chromogen Solution B | 6.0ml | 3.0ml |
| Stop Solution | 6.0ml | 3.0ml |
| Closure plate membrane | 2 | 2 |
| User manual | 1 | 1 |
| Sealed bags | 1 | 1 |

**Note:** Standard (S0 → S5) concentration was followed by:0,10,20,40,80,160 ng/mL

**Reagent preparation**

20×wash solution:Dilute with Distilled or deionized water 1:20.

**Assay procedure**

1. Prepare all reagents before starting assay procedure. It is recommended that all Standards and Samples be added in duplicate to the Microelisa Stripplate.

2. Add standard: Set Standard wells, testing sample wells. Add standard 50μl to standard well.

3. Add Sample: Add testing sample 10μl then add Sample Diluent 40μl to testing sample well; Blank well doesn’t add anyting.

4. Add 100μl of HRP-conjugate reagent to each well, cover with an adhesive strip and incubate for 60 minutes at 37°C.

5. Aspirate each well and wash, repeating the process four times for a total of five washes. Wash by filling each well with Wash Solution (400μl) using a squirt bottle, manifold dispenser or autowasher. Complete removal of liquid at each step is essential to good performance. After the last wash, remove any remaining Wash Solution by aspirating or decanting. Invert the plate and blot it against clean paper towels.

6. Add chromogen solution A 50μl and chromogen solution B 50μl to each well. Gently mix and incubate for 15 minutes at 37°C. **Protect from light.**

7. Add 50μl Stop Solution to each well. The color in the wells should change from blue to yellow. If the color in the wells is green or the color change does not

appear uniform, gently tap the plate to ensure thorough mixing.

8. Read the Optical Density (O.D.) at 450 nm using a microtiter plate reader within 15 minutes.

**Calculation of results**

1. This standard curve is used to determine the amount in an unknown sample. The standard curve is generated by plotting the average O.D. (450 nm) obtained for each of the six standard concentrations on the vertical (Y) axis versus the corresponding concentration on the horizontal (X) axis.
2. First, calculate the mean O.D. value for each standard and sample. All O.D. values, are subtracted by the mean value of the zero standard before result interpretation. Construct the standard curve using graph paper or statistical software.
3. To determine the amount in each sample, first locate the O.D. value on the Y-axis and extend a horizontal line to the standard curve. At the point of intersection, draw a vertical line to the X-axis and read the corresponding concentration.
4. Any variation in operator, pipetting and washing technique, incubation time or temperature, and kit age can cause variation in result. Each user should obtain their own standard curve.
5. The sensitivity by this assay is 1.0 ng/mL
6. Standard curve


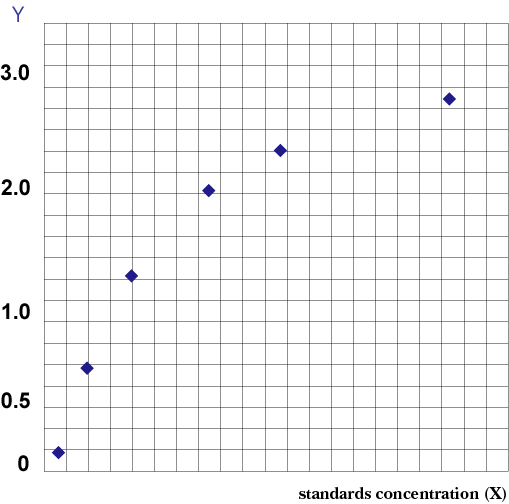


Storage： 2-8℃.

validity： six months.

**FOR RESEARCH USE ONLY; NOT FOR THERAPEUTIC OR DIAGNOSTIC APPLICATIONS! PLEASE READ THROUGH ENTIRE PROCEDURE BEFORE BEGINNING!**
