# Supplementary material for: Effect of porcine corneal stromal extract on keratocytes from SMILE‐derived lenticules
Source: J Cell Mol Med. 2020 Dec 20;25(2):1207–20. doi: 10.1111/jcmm.16189 (PMC7812260; doi:10.1111/jcmm.16189)
Supplement: Supplementary file 12 — Supplementary Material [file JCMM-25-1207-s012.docx]

Fig. S1 The hCSCs at P3 in the RIFA medium supplemented with pCSE were obviously positive to ALDH3A1 staining, whereas the hCSCs at P3 in the NM were negligibly expressed ALDH3A1. Scale bars: 50 μm.

Fig. S2 (A) GO enrichment plot of the significant biological processes potentially affected by pCSE proteins. (B) GO enrichment plot of the top 10 most notable biological processes.

Fig. S3 The results of concentration of decorin (90.65 ± 14.1 ng/ml), insulin like growth factor 2 (84.56 ± 11.7 ng/ml), and clusterin (196 ± 46.61ng/ml) verified by the ELISA assay.

Fig. S4 Mouse corneal changed by 14 days after injection. (A) Slit-lamp biomicroscopy showed that both the RIFA medium group and the RIFA medium + pCSE group corneas appeared to be transparent. (B) Immunostaining of the mouse cornea sections showed that positive CD34 and negative CD45 both in the RIFA medium group and RIFA medium supplemented with pCSE group. 50 μm for B.

Table S1 List of primers.

Table S2 [The results of nano LC-MSMS assay.](https://mc.manuscriptcentral.com/jcmm?DOWNLOAD=TRUE&PARAMS=xik_2kFrTruorESgAdACyBS7ExsMhHQDAFqDsPYMB56QTsWqrt1wZWRUHWM7sY3zZPpWn3JepG5Bbe1umPvLUqeFsXjDF7RG35B66X1c2tisYD6ae3dyKixfhR8anyTMjUkx5ojevaEH6CE7bNZc4mjR3Qwgx1GgSic3ZebdcMm1p8CXBda)

Table S3 [The top 10 most notable biological processes in GO enrichment.](https://mc.manuscriptcentral.com/jcmm?DOWNLOAD=TRUE&PARAMS=xik_4SWdVfH3AFWhmCouofBRHJD4ttt5zHwK3Y45SCkvmvwXgcQQQMjYptLzJfmSQy3KzZDyUz34GxkhyUg9oJkuLmCVviNqXwuGur8DuBdVuaCfM3WxSPHAJbBL2mJByx4P53316EGEn7koGYCuUp15Bshg19kXLkqtvojNfEvR6Zb9UiJ)
